# Genomic landscape of a metastatic malignant proliferating tricholemmal tumor and its response to PI3K inhibition

**DOI:** 10.1038/s41698-019-0077-2

**Published:** 2019-02-15

**Authors:** Jean-Nicolas Gallant, Andrew Sewell, Karinna Almodovar, Qingguo Wang, Kimberly B. Dahlman, Richard G. Abramson, Meghan E. Kapp, Brandee T. Brown, Kelli L. Boyd, Jill Gilbert, Daniel N. Cohen, Wendell G. Yarbrough, Zhongming Zhao, Christine M. Lovly

**Affiliations:** 1 Division of Hematology/Oncology, Department of Medicine, Nashville, TN USA; 2 Department of Otolaryngology, Department of Medicine, Nashville, TN USA; 3 Department of Biomedical Informatics, Department of Medicine, Nashville, TN USA; 4 Department of Radiology and Radiological Sciences, the Department of Medicine, Nashville, TN USA; 5 Department of Pathology, Microbiology, & Immunology, and the Department of Medicine, Nashville, TN USA; 6 Department of Pathology, Yale School of Medicine, New Heaven, CT USA; 7 Vanderbilt Ingram Cancer Center, Vanderbilt University Medical Center, Nashville, TN USA; 8 Present Address: Division of Otolaryngology, Department of Surgery, Yale School of Medicine, New Heaven, CT USA; 9 Present Address: Department of Computational Science, Lipscomb University, Nashville, TN USA; 10 Present Address: Department of Pathology & Immunology, Baylor College of Medicine, Houston, TX USA; 11 Present Address: The Center for Precision Health, School of Biomedical Informatics, The University of Texas Health Science Center at Houston, Houston, TX USA

## Abstract

Proliferating tricholemmal tumors (PTTs) are rare benign neoplasms that arise from the outer sheath of a hair follicle. Occasionally, these PTTs undergo malignant transformation to become malignant proliferating tricholemmal tumors (MPTTs). Little is known about the molecular alterations, malignant progression, and management of MPTTs. Here, we describe the case of a 58-year-old female that had a widely metastatic MPTT that harbored an activating *PIK3CA* mutation and was sensitive to the PI3K inhibitor, alpelisib (BYL719). We review the available literature on metastatic MPTT, detail the patient’s course, and present a whole genome analysis of this rare tumor.

## Introduction

Proliferating tricholemmal tumors (PTTs) are benign neoplasms of the external hair sheath.^1^ PTTs have the potential for malignant transformation, and, when characterized by cytologic atypia, abnormal mitoses, and infiltrating margins, are termed malignant proliferating tricholemmal tumors (MPTTs).^2^ MPTT is a rare entity, with just a few hundred cases described in the literature. While MPTT has the potential for local recurrence and metastasis, fewer than 30 cases of metastatic malignant proliferating tricholemmal tumor (that is, MPTT that has spread to or beyond regional lymph nodes) have been detailed in the literature (Table 1).^2–25^ Given the rarity of these tumors, little is known about their molecular alterations, malignant progression, and management. Aneuploidy may be common in MPTT;^26–28^ however, in depth analysis of chromosomal or structural alterations in MPTT is lacking. Here, we describe the case of a 58-year-old female that had a widely metastatic MPTT harboring an activating *PIK3CA* mutation. We detail the patient’s course and present a whole genome analysis of this rare tumor.

**Table 1:** Documented cases of metastatic MPTT

Study	Age/gender	Primary location	Sites of metastasis
Seff et al., 1916	51/M	scalp	ipsilateral cervical LN
Caylor, 1925	71/F	cheek	carcinomatosis
Peden, 1948	79/M	ear	ipsilateral cervical LN
Peden, 1948	44/M	arm	ipsilateral axillary LN
Holmes, 1968	65/F	scalp	ipsilateral periauricular LN
Saida et al., 1983	47/M	scalp	bilateral cervical LN
Amaral et al., 1984	52/F	groin	lungs, mediastinum, liver
Batman et al., 1986	70/F	scalp	ipsilateral cervical LN, accessory nerve perineum
Aricó et al., 1989	42/F	scalp	ipsilateral cervical LN
Mori et al., 1990	58/F	scalp	ipsilateral cervical LN, pleura
Sau et al., 1995	58/F	neck	ipsilateral cervical LN
Weiss et al., 1995	78/M	scalp	ipsilateral periauricular and cervical LN
Park et al., 1997	32/M	scalp	bilateral cervical LN, lungs, bone
Uchida et al., 2000	67/W	breast	axillary LN, lungs
Bae et al., 2001	32/M	scalp	brain parenchyma, lungs
Kim et al., 2001	75/M	lip	ipsilateral cervical LN
Jung et al., 2003	69/F	scalp	cervical LN
Folpe et al., 2003	55/F	back	bilateral subclavian and axillary LN, carcinomatosis
Hayashi et al., 2004	56/F	angle of jaw	bilateral cervical LN, lungs
Ye et al., 2004	66/M	scalp	regional LN, lungs, bone, liver, spleen, other skin sites
Ye et al., 2004	66/F	forehead	cervical LN, parotid gland
Siddha et al., 2007	50/F	scalp	ipsilateral cervical LN
Nakai et al., 2008	32/M	scalp	bilateral cervical para-aortic LN, lungs, liver
Eskander et al., 2010	65/F	scalp	ipsilateral cervical LN, skin, bones
Dubhashi et al., 2014	26/F	scalp	regional LN
Trikudanathan et al., 2015	65/M	scalp	ipsilateral supraclavicular LN, pancreas
Lobo et al., 2016	29/M	scalp	brain parenchyma, lungs
present case	58/F	scalp	bilateral cervical LN, accessory nerve perineum, lungs, carcinomatosis
Typical metastatic case	55/F	scalp	regional LN, lungs

*M* male, *F* female, *LN* lymph node, *carcinomatosis* widespread intra-abdominal metastases

## Results

### Case report

**Fig. 1:**
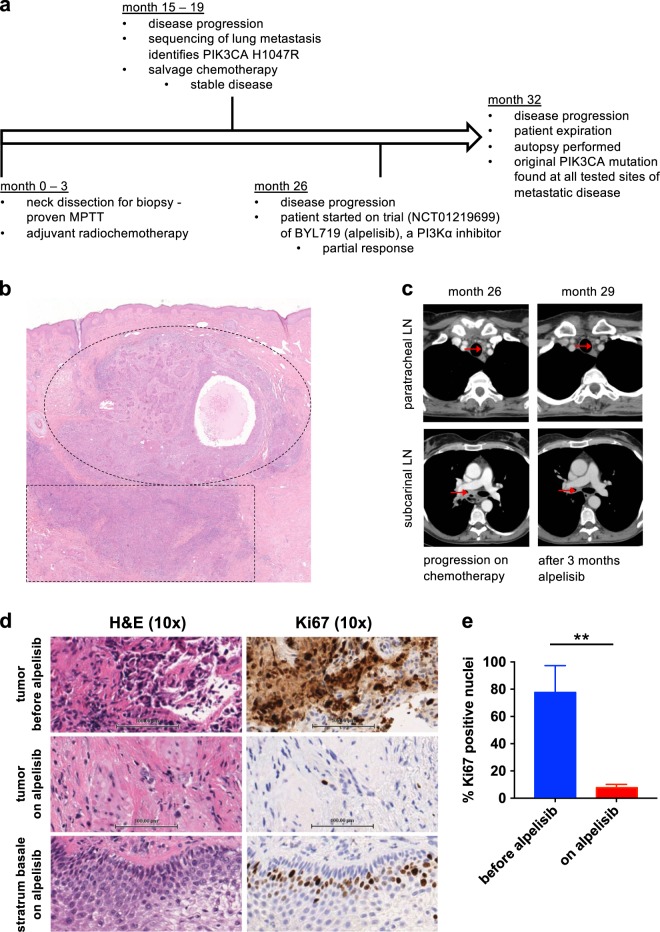
Overview of the case, including targeted response of a metastatic MPTT to PI3K inhibition. **a** Timeline of the patient’s course of disease starting with her neck dissection at a tertiary care center. Note: as detailed in the text, the patient had a recurrent posterior scalp lesion treated with local excisions for many (10+) years prior to her neck dissection. **b** Hematoxylin and eosin (H&E) stain of original diagnostic biopsy (4×). Note: (1) the dermal proliferation of convoluted lobules that infiltrate the deep dermis and subcutis, with tricholemmal type keratinization typical of PTT (dashed ellipse); and, (2) in the deeper sheets of cells there is cytologic atypia, increased mitoses, and infiltrating margins diagnostic for MPTT (dashed box). **c** CT scans of patient demonstrating radiographic response of MPTT to BYL719 (alpelisib). Left images = patient after six cycles of chemotherapy (largest paratracheal mass diameter = 13 mm; largest subcarinal mass diameter = 19 mm); right images = patient after 3 months of treatment with alpelisib (largest paratracheal mass diameter = 4 mm; largest subcarinal mass diameter = 9 mm). LN = lymph node. **d** Molecular response of MPTT to alpelisib. H&E and Ki67 (a marker of cellular proliferation) of tumor tissue before alpelisib treatment (top) and after 3 months of alpelisib treatment (middle). Nearby healthy skin (bottom) was also biopsied 3 months after initiation of alpelisib treatment and demonstrates normal proliferation of stratum basale. **e**) Quantification of tumoral Ki67 positive nuclei before and during treatment with alpelisib. Data are presented as mean ± standard deviations (*n* = 3 separate sections of ≥100 nuclei). **p = 0.0036 by unpaired *t*-test

The patient’s course is outlined in Fig. 1a.

A 58-year-old, previously healthy, white female presented to her primary care provider (PCP) with the desire to remove a right posterior scalp cyst for cosmesis. This non-inflamed, non-draining, painless, 1–2 cm cyst had been present for close to 10 years without change in size or fluctuance. The cyst was initially drained by the PCP, but, when it recurred 6 months later, the PCP excised the cyst and sent the specimen for routine pathology. The initial read of the tissue sample was high-grade invasive carcinoma with squamous features and arising in association with a PTT. Based on the pathology, the PCP referred the patient to a plastic surgeon for a more definitive excision of the lesion and repair of the defect. The lesion was excised with negative margins and pathology read as invasive high-grade squamous cell carcinoma (SCC). Eight months post resection, the lesion recurred locally along with a palpable right posterior cervical lymph node (LN). A positron emission tomography (PET) scan at that time demonstrated hypermetabolic activity in the posterior occiput and in a posterior neck LN. Fine needle aspiration (FNA) of both the primary scalp lesion and LN were completed, and pathology was reported as SCC, similar to the primary lesion.

With a working diagnosis of locally advanced SCC, the patient’s care was referred to a tertiary care center. There, a dermatopathologist re-evaluated the previous biopsy specimens and altered the diagnosis from SCC to MPTT (Fig. 1b). Subsequently, the patient was referred to a head and neck surgeon for modified radical posterior neck and lymph node dissection. Intraoperative findings uncovered the presence of nodal metastases to the posterior neck with extranodal extension, extensive perineural invasion of the spinal accessory nerve, and jugular venous invasion of the MPTT. After surgery, the case was discussed at a multi-disciplinary tumor board, and a common head and neck cancer protocol of adjuvant chemotherapy (weekly carboplatin plus paclitaxel) with concurrent radiation was recommended.^29^ The patient tolerated the adjuvant chemoradiotherapy with expected toxicities including nausea and fatigue.

Fifteen months after her neck dissection (1 year after completing chemoradiotherapy), FNA of a suspicious right paraspinal LN at C5 documented disease recurrence. The patient underwent a revision neck dissection (extending from the sternocleidomastoid, anteriorly, to halfway down the trapezius, posteriorly) with final pathology specimens consistent with metastatic MPTT. A subsequent PET scan demonstrated hypermetabolic activity in a right supraclavicular LN, multiple mediastinal LNs, and in a 0.8 × 1.1 cm right lower lung lobe parenchymal nodule. An endobronchial ultrasound (EBUS) and transbronchial needle aspiration (TBNA) showed involvement of level 4 and 7 mediastinal LN by carcinoma. Molecular profiling of the tissue using a multiplexed PCR assay (SNaPshot^30^) identified a *PIK3CA* c.G3140A (p.H1047R) mutation. Given the paucity of evidence for this mutation in this cancer, the patient was treated with a standard regimen for metastatic SCC (consisting of docetaxel and cisplatin every three weeks).^31^ At the same time, the patient was put on the waitlist for NCT01219699, a phase I study of oral BYL719 (alpelisib, a PI3Kα-selective inhibitor) in adult patients with advanced solid malignancies, whose tumors have an alteration of the *PIK3CA* gene.^32^

Seven months after completion of her second chemotherapy regimen, the patient was found to have progressive metastatic pulmonary disease on routine computerized tomography (CT) scan (she was asymptomatic at this time). The patient then enrolled in the trial of alpelisib at 450 mg daily. Her only suspected adverse effects related to the study drug were nausea and weight loss. After 3 months of treatment, she demonstrated a partial response per RECIST (Fig. 1c and S1).^32,33^ Additionally, an on-treatment research biopsy, obtained 3 months after the start of alpelisib, demonstrated a significant reduction in proliferation as assessed by Ki67 staining (Fig. 1d, e).

Four months after starting the study drug, the patient developed a community acquired pneumonia (CAP), and the treatment was suspended. Although the CAP resolved both clinically and radiographically, the patient was started on 2 L/min oxygen due to a persistent cough that was not responsive to therapy. It was unclear if the patient had progressive disease or a drug-related pneumonitis at this time (although this is not a known adverse effect of alpelisib^32^). Two months after stopping alpelisib, a surveillance CT scan demonstrated peritoneal carcinomatosis. Within days of this CT scan, the patient was admitted to the hospital with increased work of breathing requiring 4 L/min oxygen. The patient opted for no aggressive measures and was symptomatically treated until her death a week later.

Prior to her passing, the patient consented to a rapid autopsy, which revealed the suspected cause of death to be widely metastatic MPTT. There were innumerable metastases in the lungs, the largest being 3.0 × 3.0 × 2.2 cm in dimension (Fig. S2a). Another heavily involved site was the liver, which contained multiple metastases, the largest being 3.5 × 2.5 × 0.6 cm in dimension (Fig. S2b). The cancer also was found to involve a papillary muscle of the heart, the pericardium, fundus of the stomach, ileum, colon, omentum, right ovary, soft tissues of the neck, both adrenal glands, and the pelvic peritoneum. Interestingly, many of the lesions identified at autopsy were not visualized on the CT scan taken two weeks prior to the patient’s death. Neuropathology demonstrated no evidence of CNS metastases. No microbiological evidence of pneumonia was found in the lungs, and cultures did not grow any causative pathogenic bacteria or fungi.

### Genomic characterization of a MPTT

**Fig. 2:**
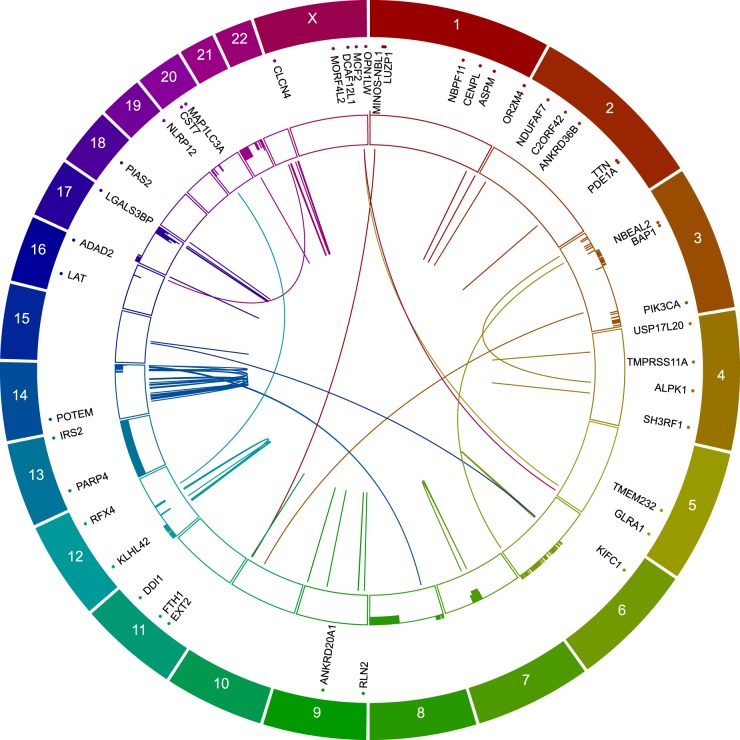
Genomic characterization of a metastatic MPTT. CIRCOS^83^-style plot demonstrating the position of mutations, CNVs, and SVs found in a metastatic MPTT by WGS. Each chromosome is delineated by the appropriate letter or number and the corresponding color-matched segments of the concentric rings. The segments of the outermost ring represent the normal relative length along/of each chromosome. Points and text along each of the chromosomes indicate the position of the 43 non-silent coding mutations identified in the tumor. The segments of the inner ring represent CNVs along the length of each chromosome. The outer boundary of these segments is the baseline copy number of 2 (diploid), and the inner boundary is scaled to a copy number of 10. Bars in each of these segments indicate copy number loss or gain, with the size and direction of the bar correlating with the magnitude of gain or loss. The center of the plot displays any/all SVs, including inter and intra-chromosomal translocations, insertions, and deletions

To gain a better understanding of the molecular alterations that may drive MPTT, we performed whole genome sequencing (WGS) of the tumor. First, we verified that the numerous cancerous lesions found at autopsy were all metastases from the same cancer and not secondary cancers. Ten of the lesions that were sampled during autopsy were molecularly profiled with the SNaPshot assay,^30^ and each found to harbor the PIK3CA H1047R mutation (data not shown). Second, we performed WGS on the primary tumor and compared it to normal liver tissue from the patient. The WGS coverage was 29.3× for the normal and 29.0× for the tumor samples. Using a robust analysis pipeline^34^ (Fig. S3), we identified numerous somatic exonic, copy number, and structural variants (Fig. 2). Average Phred quality scores were above 30 (>99.9% base call accuracy) for all mutational calls (Fig. S4).

Using our WGS analysis pipeline, 18,120 somatic variants (single nucleotide variants [SNVs] and small insertions and deletions [indels]) were detected, including 6139 germline variants already reported in the dbSNP (v137)^35^ database (Table S1). Most of these somatic mutations were in intergenic (7171), intronic (4125), or non-coding sequences (e.g., ncRNAs, UTRs); but, we identified of 43 non-synonymous mutations in the tumor genome (Fig. 2 and Table S2). Among them, 38 were not reported in the Catalogue of Somatic Mutations in Cancer (COSMIC),^36^ and 2 were predicted to be cancer-causing or driver mutations by the Functional Analysis Through Hidden Markov Models (FATHMM)^37^ tool—*ANKRD20A1* c.G599A (p.R200Q) and *DCAF12L1* c.G1366T (p.G456W). As a validation of our methods, PIK3CA H1047R was identified by our WGS pipeline and predicted to be cancer-causing per FATHMM. Targeted dideoxy sequencing was performed on 1/3 of the mutations to corroborate the SNV calls, and each of the mutations was validated exclusively in tumor tissue (Fig. S5).

Our analysis also uncovered a variety of somatic copy number variations (CNVs) and structural variants (SVs). Specifically, we identified 238 CNVs covering 1,495 unique genes (Fig. 2 and Table S3). At the chromosomal arm level, this included significant losses in 3p, 8p, 6q, and 17p along with gains in 3q, 7q, 14q, 17q, 22q, and the entirety of 13. The most significant (z-scored) focal CNVs were those of *DNAH17* and *PGS1*, which were each amplified to 8 copies. We also identified 59 SVs spanning 662 unique genes (Fig. 2 and Table S4). Most of these SVs were intra-chromosomal translocations (*n* = 23), and the rest were equally divided amongst inter-chromosomal translocations (*n* = 11), insertions (*n* = 12), and deletions (*n* = 13). None of the SVs discovered in our WGS samples appear to be relevant for MPTT oncogenesis or metastasis. However, further examination of gene or protein expression could help determine if a tumor relationship exists.

To learn more about the list of altered genes in metastatic MPTT, we performed a variety of enrichment analyses to determine whether specific altered genes were over/under represented in the metastatic MPTT. No single pathway, ontology, phenotype, or cell type was significantly over-represented in our analyses (data not shown). Moreover, the altered genes in metastatic MPTT did not significantly overlap with genes associated with the outer root sheath/bulb of hair (the putative origin of MPTT^1^) or with those altered in cutaneous SCC (a much more common and histologically similar malignancy) (Table S6). Finally, we did not detect a UV signature in the genome of this tumor, which is notable since the primary lesion was in a sun exposed area.

## Discussion

MPTT is a rare entity, with just a few hundred cases described in the literature. However, the true prevalence of the cancer may be obscured by its common misdiagnosis as SCC and by its abundance of alternative names.^12^ MPTT is difficult to distinguish from SCC due to its shared behavior (invasion), common location (scalp), and overlapping histology—including the potential presence of spindle cells.^23,38^ However, SCC is classically heralded by actinic keratoses, and histologic evidence of keratin production suggests SCC. Meanwhile evidence of tricholemmal keratinization, a lobular pattern (Fig. 1b), and reactivity with AE13 and AE14 suggests MPTT.^39,40^ Establishing a proper diagnosis is key as cutaneous SCC is a common and well-studied disease with established treatment algorithms,^41^ whereas MPTT remains unstudied and unpredictable. At the same time, MPTT—which, among others, also has been called malignant proliferating epidermoid cyst, pilar tumor, tricholemmal cyst, invasive pilomatricoma, trichochylamydocarcinoma, proliferating isthmic cystic carcinoma, and giant hair matrix tumor—should not be confused with other adnexal tumors, such as tricholemmal carcinoma (a form of malignant tricholemmoma), as these are known to occur on the scalp but not to metastasize.^1^ Our sequencing data hint that cutaneous SCC and MPTT are genetically distinct and could potentially be differentiated with a gene signature. Moreover, due to the low prevalence of *PIK3CA* mutations in cutaneous SCC,^42,43^ the malignancy has the potential to be differentiated with a simple gene (*PIK3CA*) test. However, more data are needed to confirm this hypothesis. For now, consultation with a board-certified dermatopathologist is recommended if a diagnosis of unusual squamous cell carcinoma or MPTT is considered.

We performed an extensive literature review and uncovered all known cases of metastatic MPTT (Table 1). While MPTT is known to metastasize, due to the limited number of cases of metastatic MPTT, little is known about its malignant progression. It is generally assumed that benign tricholemmal cysts very rarely undergo step-wise malignant transformation—starting with the adenomatous stage of the tricholemmal cyst through the epitheliomatous stage of the PTT—into carcinomatous MPTTs.^7^ However, it is unclear how this process unfolds and/or how often MPTT metastasizes. Categorization of the process has been attempted based on histomorphology;^2^ however, cases with little or no cytologic and architectural atypia may exhibit aggressive behavior and vice-versa.^26,44^ It is surprising that MPTT does not metastasize more often given the immune privilege of cells in the hair shaft; one would expect that hair shaft tumors (secondary to their low level of major histocompatibility molecules) would more readily metastasize.^45^ More studies are needed to understand the biology and clinical course of MPTT, especially with regards to biomarkers that may predict disease severity.

There have been just a handful of investigations into the molecular alterations of MPTT. Early studies identified and focused on the high percentage of aneuploid cells found in MPTT.^26,46^ Later studies concentrated on the elevated proliferation rate of these malignancies as demonstrated by Ki67 immunostaining.^27,47^ To date, only two studies have examined the genetics of these tumors, and both focused on *TP53* mutations.^22,48^ Genetic analysis of this patient’s MPTT did not reveal any alterations in *TP53*.

Our WGS of a metastatic MPTT instead identified numerous exonic and structural variants (Fig. 2 and Tables S2–4) and PIK3CA H1047R as the only known driver mutation. We otherwise identified 42 mutations, 238 CNVs, and 59 SVs. The mean burden of 1.5 mutations per megabase (1.5 mut/Mb) puts MPTT at about the average mutational burden among all the major cancer types examined.^49–51^ Interestingly, the amount of structural alterations, including focal and chromosome-arm-level CNVs, is well above the average of most tumors types.^52^ While the structural alterations did not reveal any obvious fusion, amplification, or driving event, analysis of the mutations found in MPTT uncovered two potentially cancer causing mutations—ANKRD20A1 R200Q and DCAF12L1 G456W. ANKRD20A1, a poorly studied ankyrin family member, is thought to be involved in mediating protein-protein interactions but has only been studied as a biomarker in Behçet’s disease.^53^ DCAF12L1 is involved in germ cell development and is linked to extracellular signal-regulated kinase signaling.^54^ Interestingly, both of these genes’ products are normally expressed only in ciliated tissues per the human protein atlas.^55^ As with any genetic study, more laboratory work is needed to validate any potential functional significance of these mutations.

Given that the outer root sheath is a proliferative compartment, it is surprising that not many of these genes were altered or deregulated. In contrast to similar proliferative compartments such as the gut or bone marrow, the hair follicle appears to have evolved a striking resistance to oncogenic stimuli, either because of a relatively protected anatomic location, unknown biologic factors, or combination of the two.^1^ Interestingly, the hair shaft is driven by Wnt signaling, and alterations in this pathway lead to a distinct type of tumor: pilomatricoma.^56^ No genes linked with Wnt signaling were altered in MPTT. Likewise, the similar cutaneous SCC often arises from alterations in Notch signaling^42,43^ and none of these mediators were altered in MPTT. The difference in genetics between MPTT, cutaneous SCC, and the normal hair shaft suggests the existence of an alternate mechanism of oncogenesis. This finding also challenges the dogma that MPTT arises from the outer root of the hair shaft.^1^ More MPTTs should undergo genomic profiling to uncover this pathway and help treat patients.

Due to its poorly understood biological behavior, local excision with wide margins (≥2 cm) is the most common primary treatment for MPTT in the literature. However, given the relatively high local recurrence rate (4–7%) with a median literature follow-up of 15 months, other surgical treatment modalities are being investigated.^2^ Mohs surgery and excision with frozen section margin assessment is an intriguing emerging option as it has the benefit of decreasing the rate of recurrence while simultaneously sparing tissue in larger lesions.^57^ However, as this case highlights, early involvement of a head and neck surgeon may be beneficial in cases of biopsy-proven MPTT due to their tendency for locoregional spread and cervical LN metastasis.

There is a paucity of information on the non-surgical management of MPTT (Table 2).^2,6–9,12,13,15,16,20–24,44,58–61^ The few studies that do exist have shown: that ethanol injections may be useful for local recurrence; that chemotherapy regimens containing cisplatin, doxorubicin, and vinca alkaloids can be beneficial for metastatic disease; and that radiotherapy can be effective in preventing primary lesion recurrence. Here, we show that metastatic MPTT can be responsive to a variety of chemotherapy regimens and a small molecule inhibitor of PI3K, in the setting of a somatic *PIK3CA* activating mutation (Fig. 1c).^32^ This case highlights the power of precision oncology, integrated genomics, and targeted agents.^62^ As recent studies have shown ≥ 50% objective response rates when rare tumors are sequenced and actionable mutations matched to available therapies,^63^ it may be reasonable to include mutational testing and targeted therapy as part of the workup and treatment of MPTT.

**Table 2:** Documented non-surgical treatment regimens used against MPTT

Study	Non-surgical treatments	Response
Holmes et al., 1968	Radiotherapy	Regression of the 1º tumor; progression of metastases
Saida et al., 1983	270 mg bleomycin after each surgery	Stable disease for 24 months
Amaral et al., 1984	Radiotherapy to a total dose of 50 Gy	No 1º tumor recurrence; progression of metastases
Batman et al., 1986	Radiotherapy	1º tumor recurrence; metastases
Weiss et al., 1995	Neoadjuvant cisplatin and fluorouracil	Reduction in 1º tumor size
Sau et al., 1995	Chemotherapy and radiotherapy	N/A
Noto et al., 1997	(1) Radiotherapy	(1) 1º tumor recurrence; metastases
	(2) Cisplatin, alpha-interferon, and vinorelbine	(2) Locoregional extension of the 1º tumor
Takenaka et al., 1998	(1) Chemotherapy (cisplatin, bleomycin), radiotherapy (to a total dose of 60 Gy), and hyperthermia	(1) 1º tumor recurrence after 3 years
	(2) Chemotherapy (cisplatin, doxorubicin) for 4 months	(2) 1º tumor progression; invasion into the cranium
	(3) Weekly intratumoral ethanol injection (15 mL)	(3) Stable disease for 18 months
Yoleri et al., 1999	Radiotherapy	N/A
Uchida et al., 2000	Cyclophosphamide, doxorubicin, 5-fluorouracil, mitomycin C, and fadrozol*	Stable disease for 6 months
Bae et al., 2001	75 mg/m2 cisplatin on day 1 and 100 mg/m2 etoposide on days 1–3 for one cycle	Partial response for 6 months
Hayashi et al., 2004	150 mg cisplatin, 50 mg doxorubicin, and 3 mg vindesine given over 3 days and repeated every 6 weeks	Locoregional extension of the 1º tumor
Ye at al., 2004	Radiotherapy	No 1º tumor recurrence
Siddha et al., 2007	Radiotherapy at 2.0 Gy per day, 5 days per week for 7 weeks, to a total dose of 60 Gy	N/A
Nakai et al., 2008	(1) Neoadjuvant 5 mg peplomycin daily for 5 days and one injection of 6 mg of mitomycin C before surgery	(1) N/A
	(2) 10 mg doxorubicin and 20 mg cisplatin daily for 4 days and radiotherapy to a total dose of 60 Gy	(2) no 1º tumor recurrence; progression of metastases
Eskander et al., 2010	Radiotherapy to a total dose of 70 Gy	Metastases within 2 months of treatment end
Dubhashi et al., 2014	Radiotherapy at 2.0 Gy per day, over the course of 21 days during one month, to a total dose of 42 Gy	No 1º tumor recurrence
Sutherland et al., 2017	Neoadjuvant radiotherapy to a total of 45 Gy delivered over 3 weeks	Complete response of the 1º tumor, obviating the need for surgery
present study	(1) 30 mg/m2 paclitaxel and 1 mg/mL•min (AUC = 1) carboplatin weekly for 7 weeks and radiotherapy at 1.8 Gy per day, 5 days per week, to a total dose of 70.2 Gy	(1) Stable disease for 12 months
	(2) 75 mg/m2 docetaxel and 75 mg/m2 cisplatin every three weeks for 6 weeks	(2) Stable disease for 4 months
	3) 450 mg alpelisib daily (in the presence of a PIK3CA mutation)	(3) Partial response for 4 months; progression of metastases

Only treatments meant to be definitive (not palliative) are included. Full treatment information is provided as detailed in the referenced publications. Unless otherwise indicated, all treatments were given as adjuvants / following primary tumor resection. Numbers indicate the order of treatment regimens

*N/A* not applicable (tumor response not measured or noted), *1°* primary, *Gy* = gray;

* = tumor initially diagnosed as a breast cancer and treated as such

In conclusion, MPTT is a rare cancer with an ill-defined but considerable metastatic potential. The identification of a targetable PIK3CA mutation in a patient with MPTT sheds light on the biology of this tumor and increases the non-surgical options for the management of this poorly-studied disease.

## Methods

### Study oversight

All patient biopsy samples were obtained under Vanderbilt University Medical Center Institutional Review Board (IRB)–approved protocols. Specifically, tissue was collected for study through the Head and Neck Cancer Tissue Repository and Clinical Database (IRB #030062). Written informed consent was obtained from the patient. All samples were de-identified, protected health information reviewed according to the Health Insurance Portability and Accountability Act (HIPAA) guidelines, and studies conducted in accordance with the Declaration of Helsinki. The patient consented to be treated with BYL719 (alpelisib) under NCT01219699, a phase I study of oral alpelisib (a PI3Kα inhibitor) in adult patients with advanced solid malignancies (Vanderbilt CTSR #PHI-1065). The patient’s spouse consented for a full hospital autopsy without limits, allowing for any tissue, organs, or fluids that are removed from the body to be studied, disposed of, or used for teaching and research. All witnessed and signed consent forms are on file.

### Immunohistochemistry of patient samples

Immunohistochemistry (IHC) of patient samples was performed at the Vanderbilt Translational Pathology Shared Resource core laboratory. Formalin-fixed paraffin-embedded tissue was used for all immunohistochemistry. For hematoxylin and eosin (H&E) staining, slides were prepared using an automated H&E stainer (Gemini) using the manufacturer’s protocol and customary dyes (hematoxylin ref#7211; eosin ref#7111; Richard-Allen Scientific). Ki67 staining was prepared using an automated IHC slide stainer (BOND Max, Leica) with slight deviations from the manufacturer’s protocol: antigen retrieval was performed at a pH of 9 for 20 minutes; slides were incubated with Ready-To-Use anti-Ki67 (pre-diluted antibody, PA0230, Leica) for 60 minutes; and BOND Polymer Refine detection system (Leica) was used for visualization. Both H&E and IHC slides were digitized using a light microscope and camera (Nikon).

Quantification of Ki67 positive nuclei was performed by a trained pathologist. Briefly, 10x digitized images (stained, as detailed above) containing at least 100 nuclei were manually counted for Ki67 positivity. This was repeated three times (using different sections of tumor) for both pre- and post- PI3K treatment. Data were plotted with Graphpad Prism 7 and significance of difference determined using an unpaired t-test.

### Sample processing and DNA extraction

Samples were processed per the Vanderbilt Head and Neck Cancer Tissue Repository and Clinical Database standards operating procedures. Surgical specimens were flash frozen in liquid nitrogen. Following H&E staining for tumor content, the freshly frozen tissue then was macro-dissected to enrich for ≥70% tumor content by a trained histotechnologist. Ten 30 µM slices of enriched tumor tissue were subsequently transferred to a microcentrifuge tube. Genomic DNA was extracted from this tube using standard proteinase K digestion and phenol extraction. Briefly, tumor tissue was incubated in DNA extraction buffer (50 mM Tris, pH8.0, 100 mM EDTA, 100 mM NaCl, 1% SDS) and proteinase K overnight at 56 °C. Lysates were then treated with RNase A for 1 h at 37 °C, phenol:choloroform extracted, and DNA precipitated using isopropanol. The resulting gDNA was run on an agarose gel.

### DNA library preparation and whole genome sequencing

DNA library preparation and whole genome sequencing was performed by the Vanderbilt Technologies for Advanced Genomics (VANTAGE) core laboratory. Briefly, 200 ng of intact genomic DNA was fragmented to an average size of 300 bp on a LE220 focused ultrasonicator (Covaris). Fragmented DNA was used to generate sequencing-ready libraries with indexed adaptors (Illumina). Library quality was assessed using the 2100 Bioanalyzer (Agilent) and libraries were quantitated using KAPA Library Quantification Kits (KAPA Biosystems). Pooled libraries were subjected to 100 bp paired-end sequencing on a HiSeq2500 (Illumina) per the manufacturer’s protocol. CASAVA v1.8 (Illumina) was used to generate de-multiplexed FASTQ files.

### Data processing and alignment

For an overview of the analysis pipeline, see Figure S3. Briefly, raw reads in FASTQ format where input into FASTQC v0.10.1^64^ to check for sequence quality (Fig. S5). Reads were then mapped onto GRCh37/hg19 using Burrows-Wheeler Aligner v0.7.1.^65^ BAM format files of the results were created with SAMtools v0.1.19.^66^ Duplicate reads were removed using Picard MarkDuplicates tool v1.75.^67^ Next, to improve SNP and indel detection, the aligned reads were realigned and base quality score recalibrated following the Genome Analysis Toolkit v2.5-2 best practices recommendations.^68,69^ The analysis-ready reads from a pair of normal and tumor tissues were then used to call mutations (single nucleotide variants (SNVs) and indels) unique to the tumor tissue using VarScan v2.3.5,^70^ MuTect v1.1.3,^71^ and Strelka v1.0.6^72^ using default parameters. A mutation was preserved if <2 reads supported the variant allele in the normal sample, if its average Phred quality score was >30, if it was not a strand bias artifact, and if it was not included in dbSNP v137.^35^ Tumor specific copy number variants were detected using Control-FREEC^73^ in its default settings. Somatic structural variants were characterized from the analysis-ready alignment files using tool CREST v1.1^74^ with default parameters. Variant annotation was performed using ANNOVAR v2013-5-9.^75^

### Targeted dideoxy sequencing for SNV confirmation

As detailed above, DNA was extracted from freshly frozen tissue sections using the DNeasy Blood & Tissue Kit (Qiagen). PCR was performed with HotStarTaq Master Mix (Qiagen) using 50 ng DNA and the primers in Table S5 at 1 µM. Amplicons were sequenced bi-directionally using M13 primers, BigDye Terminator chemistry, and 3730 DNA Analyzers (Applied Biosystems). Sequence tracings were manually aligned, verified, and cropped into Figure S6.

### Functional gene set enrichment analyses

Genes known to be significantly associated with the outer root sheath/bulb of hair were compiled from previously published studies^76–79^ and, if necessary, converted to human homologs using biomaRt.^80^ Similarly, genes known to be mutated in cutaneous squamous cell carcinomas were compiled from previous studies.^42,43^ These sets of genes (Table S6) were then compared to all genes altered in the metastatic MPTT, including SNVs, CNVs, and SVs, using hypergeometric probability. For enrichment analyses, unranked gene lists (SNVs, CNVs, SVs, and combinations thereof) were input into Enrichr v7.18^81,82^ and analyzed for significant associations.

### Literature review

A comprehensive literature review was performed using the PubMed/MEDLINE database. The literature review was completed in July 2017 using the following search strategy: ((malignant[tiab] OR metas*[tiab] OR tumor[tiab]) AND (tricholemmal[tiab] OR trichilemmal[tiab] OR pilar[tiab] OR hair shaft[tiab] OR hair matrix[tiab] OR hair follicle[tiab]). Studies from all available dates were included in the search, but language was limited to English language studies only. The database search yielded 930 results linked to the search query. Titles and abstracts were screened for further study based on their description of metastatic MPTT, including its molecular alterations, malignant progression, and management. Full text review of pertinent articles contributed the data for Tables 1–2.

### Reporting Summary

Further information on research design is available in the Nature Research Reporting Summary.

## Supplementary information

Supplementary Appendix

Reporting Summary

## Data Availability

Most data generated or analyzed during this study are included in this published article (and its supplementary appendix). Additional raw data generated during the current study are available from the corresponding authors on upon request.

## Supplementary information

**Supplementary information** accompanies the paper on the *npj Precision Oncology* website (10.1038/s41698-019-0077-2).
