# Supplementary material for: Genomic landscape of a metastatic malignant proliferating tricholemmal tumor and its response to PI3K inhibition
Source: NPJ Precis Oncol. 2019 Feb 15;3:5. doi: 10.1038/s41698-019-0077-2 (PMC6377617; doi:10.1038/s41698-019-0077-2)
Supplement: Supplementary file 1 — Supplementary Appendix [file 41698_2019_77_MOESM1_ESM.pdf]

Supplementary Appendix for:

Genomic landscape of a metastatic malignant proliferating tricholemmal tumor and its response to PI3K inhibition

Jean-Nicolas Gallant[1,*], Andrew Sewell[2,7,*], Karinna Almodovar[1,*], Qingguo Wang[3,8,*], Kimberly B. Dahlman[1], Richard G. Abramson[4], Meghan E. Kapp[5], Brandee T. Brown[2], Kelli L. Boyd[5], Jill Gilbert[1,†], Daniel N. Cohen[5,9], Wendell G. Yarbrough[2,8,10,†], Zhongming Zhao[3,6,11,†], and Christine M. Lovly[1,6,†,‡]

From the:
[1]Division of Hematology/Oncology, Department of Medicine, [2]Department of Otolaryngology, [3]Department of Biomedical Informatics, [4]Department of Radiology and Radiological Sciences, the [5]Department of Pathology, Microbiology, & Immunology, and the [6]Vanderbilt Ingram Cancer Center, Vanderbilt University Medical Center

Present addresses:
[7]Division of Otolaryngology, Department of Surgery, Yale School of Medicine
[8]Department of Computational Science, Lipscomb University
[9]Department of Pathology & Immunology, Baylor College of Medicine
[10]Department of Pathology, Yale School of Medicine
[11]The Center for Precision Health, School of Biomedical Informatics, The University of Texas Health Science Center at Houston.

*co-first authors
[†]co-senior authors

[‡]corresponding author

Contents:
Supplementary Figures and Legends 2
Supplementary Tables and Legends..13

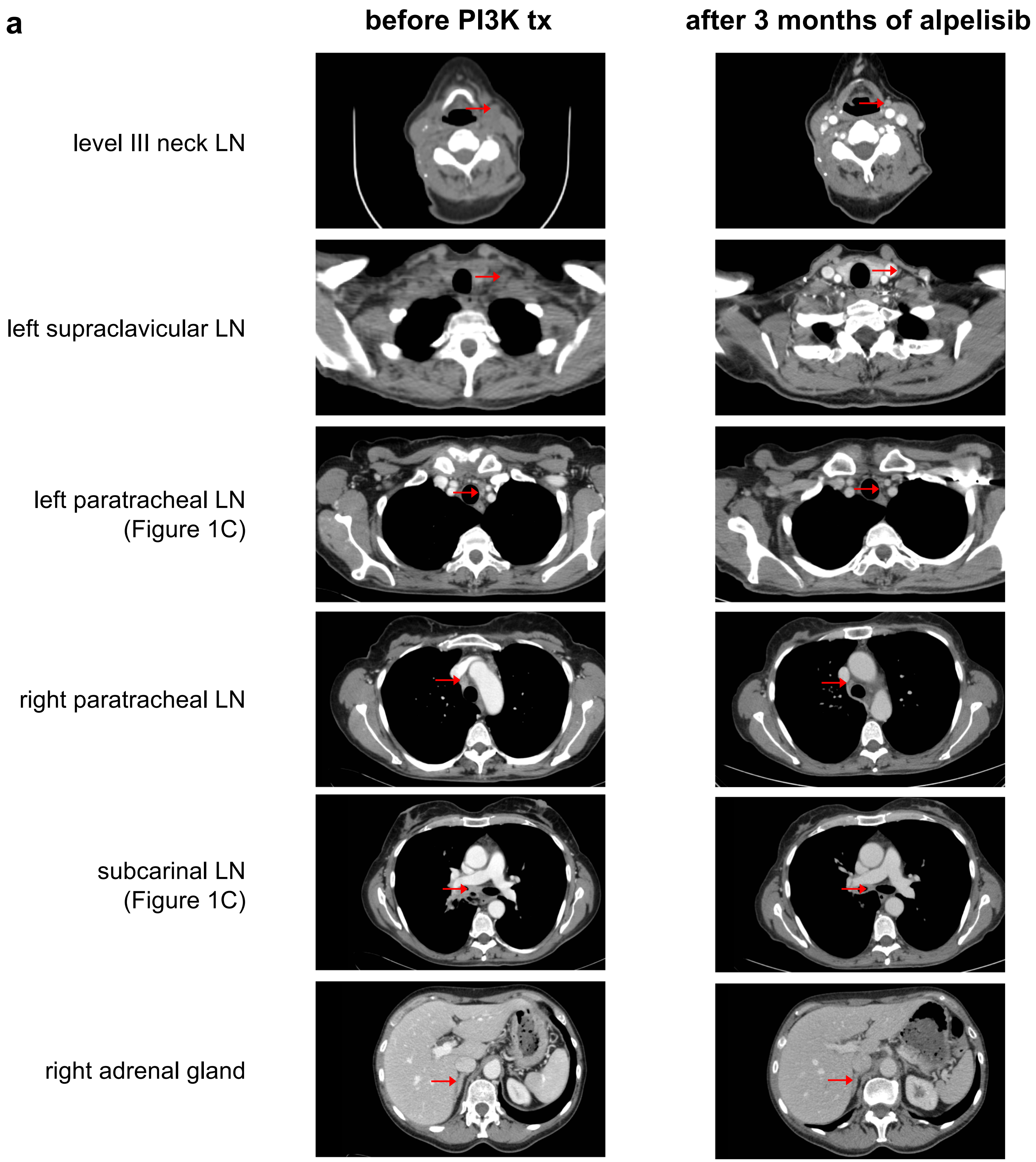

Figure S1 p1/2

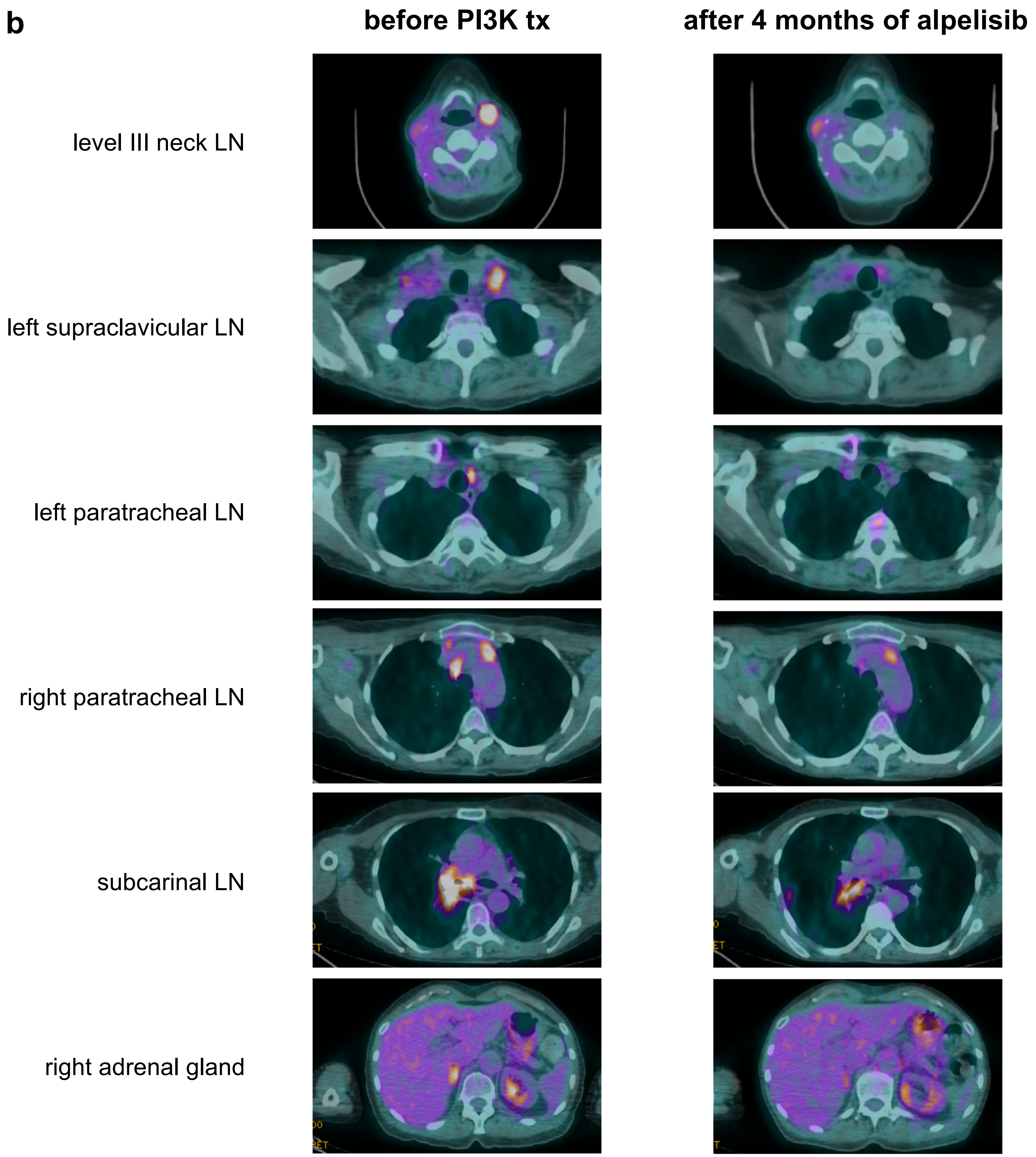

Figure S1 p2/2

Figure S1: Radiographic response to PI3K inhibition in a patient with a metastatic MPTT.
Serial CT (**a**) and PET (**b**) scans, from a patient with a MPTT harboring a PIK3CA H1047R mutation, documenting a response to the PI3Kα inhibitor, BYL719 (alpelisib). This figure includes the images from Figure 1C as well as additional images showing response to alpelisib at other disease sites. Red arrows demonstrate the lesions used for RECIST measurements; tx = treatment; LN = lymph node.

a

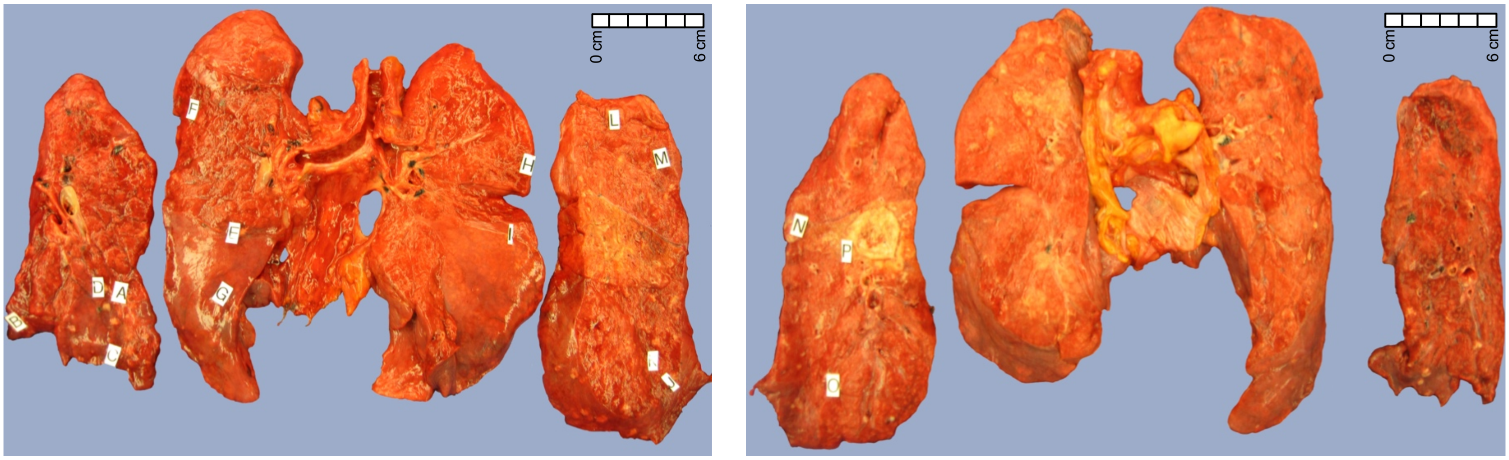

b

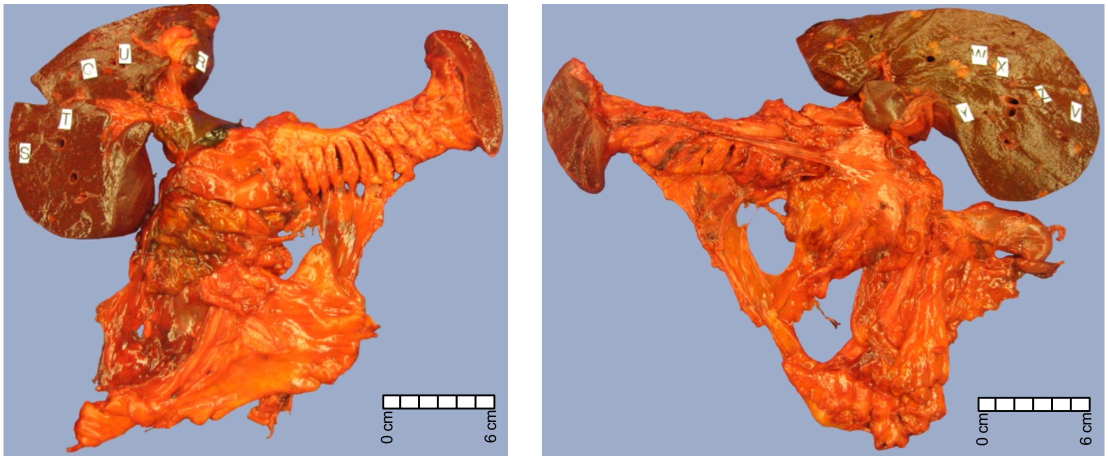

Figure S2

Figure S2: Post-mortem autopsy demonstrating widespread MPTT metastases.
Anterior (left) and posterior (right) views of the lungs (**a**) and liver, stomach, and spleen (**b**) from post-mortem autopsy demonstrating widespread MPTT metastases. Palpable and measurable metastases are marked A–Z. 6 cm rulers digitized for scale. The largest lesion in the lung is marked as ‘P’ and measured 3.0 x 3.0 x 2.2 cm. The largest lesion in the liver is marked as ‘R’ and measured 3.5 x 2.5 x 0.6 cm. Not pictured: metastases involving the papillary muscle of the heart, the pericardium, the fundus of the stomach, the ileum, the colon, the omentum, the right ovary, the soft tissues of the neck, both adrenal glands, and the pelvic peritoneum.

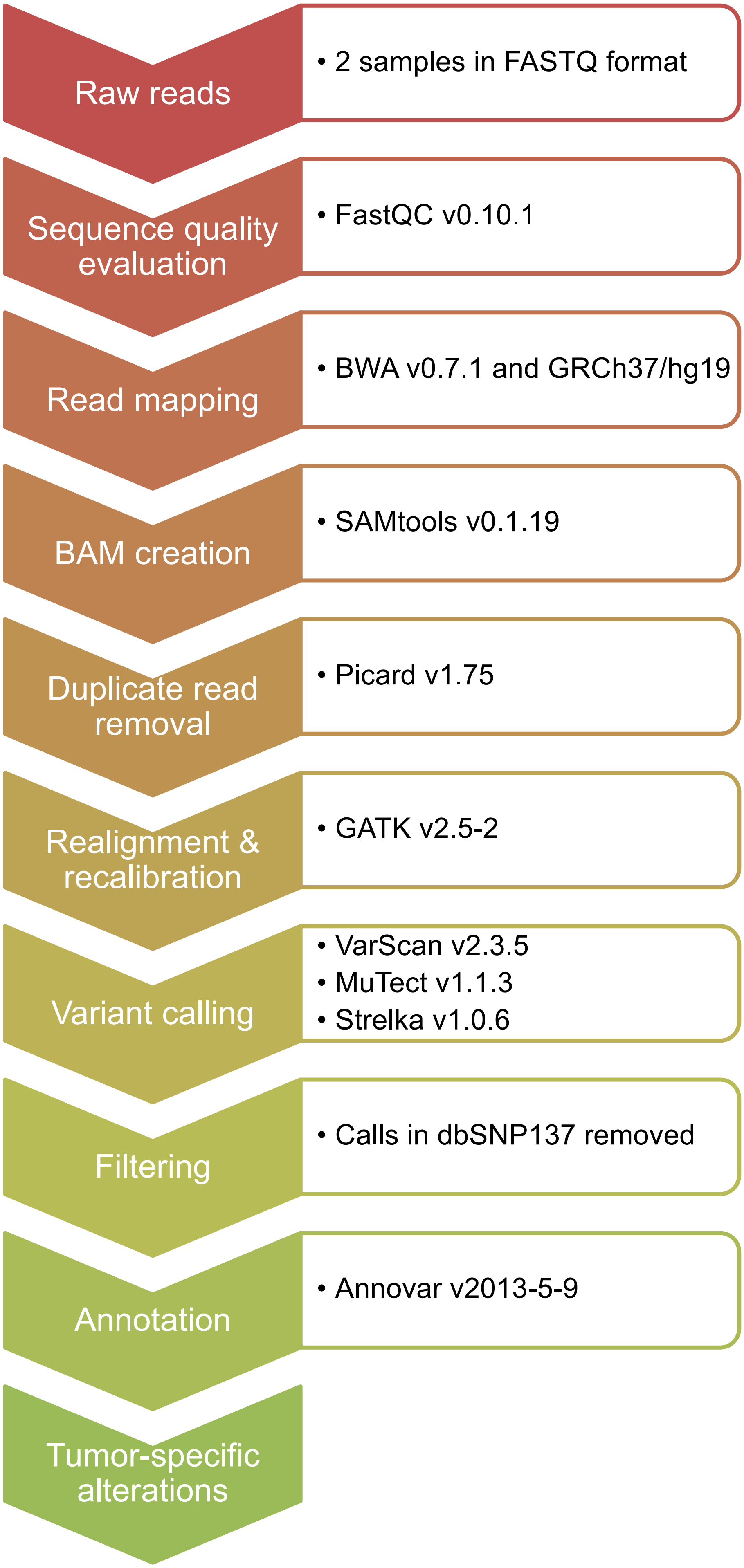
Raw reads
• 2 samples in FASTQ format
Sequence quality evaluation
• FastQC v0.10.1
Read mapping
• BWA v0.7.1 and GRCh37/hg19
BAM creation
• SAMtools v0.1.19
Duplicate read removal
• Picard v1.75
Realignment & recalibration
• GATK v2.5-2
Variant calling
• VarScan v2.3.5
• MuTect v1.1.3
• Strelka v1.0.6
Filtering
• Calls in dbSNP137 removed
Annotation
• Annovar v2013-5-9
Tumor-specific alterations

Figure S3

Figure S3: Overview of data processing pipeline.
Overview of the whole genome sequencing analysis pipeline starting from paired raw FASTQ sequencing files and ending with a dataset of tumor-specific alterations. For details about each step and tool in the process, see Methods.

a

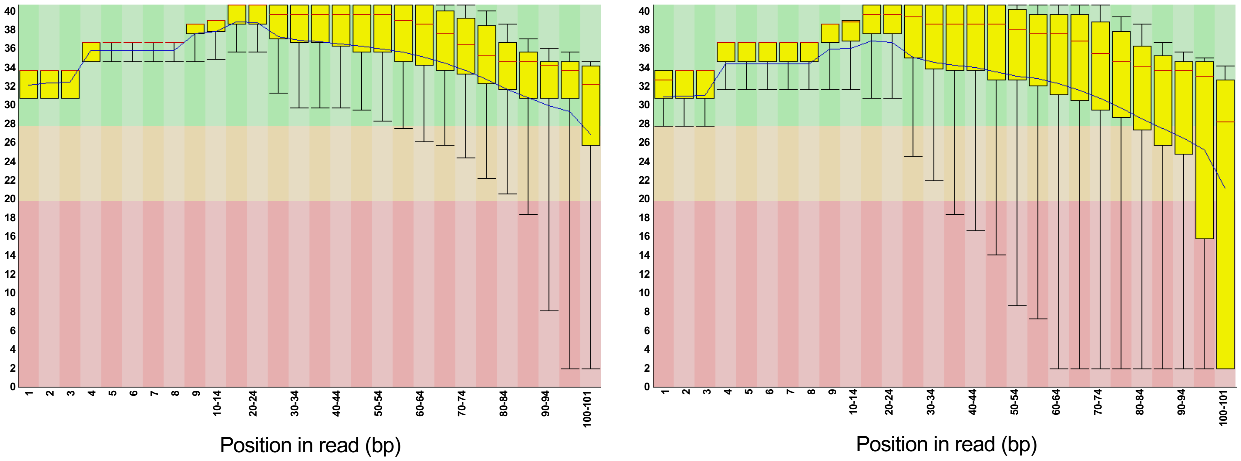
Position in read (bp)
Position in read (bp)

b

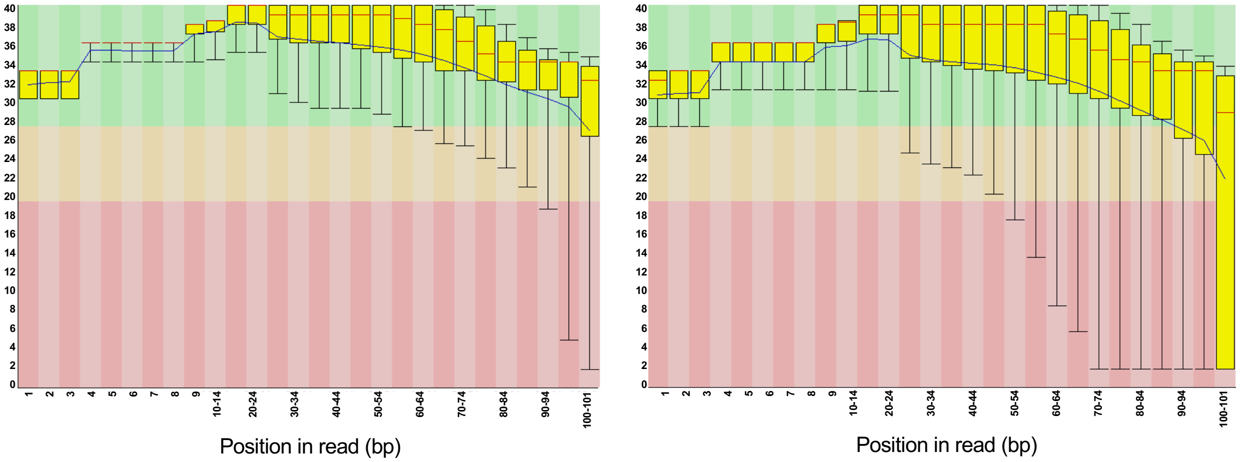
Position in read (bp)
Position in read (bp)

Figure S4

Figure S4: Per base quality of whole genome sequencing data.
Average per base quality of reads in the raw sequencing files from normal (**a**) and tumor (**b**) tissue. The left panel is the first read of the data and right panel the second read.

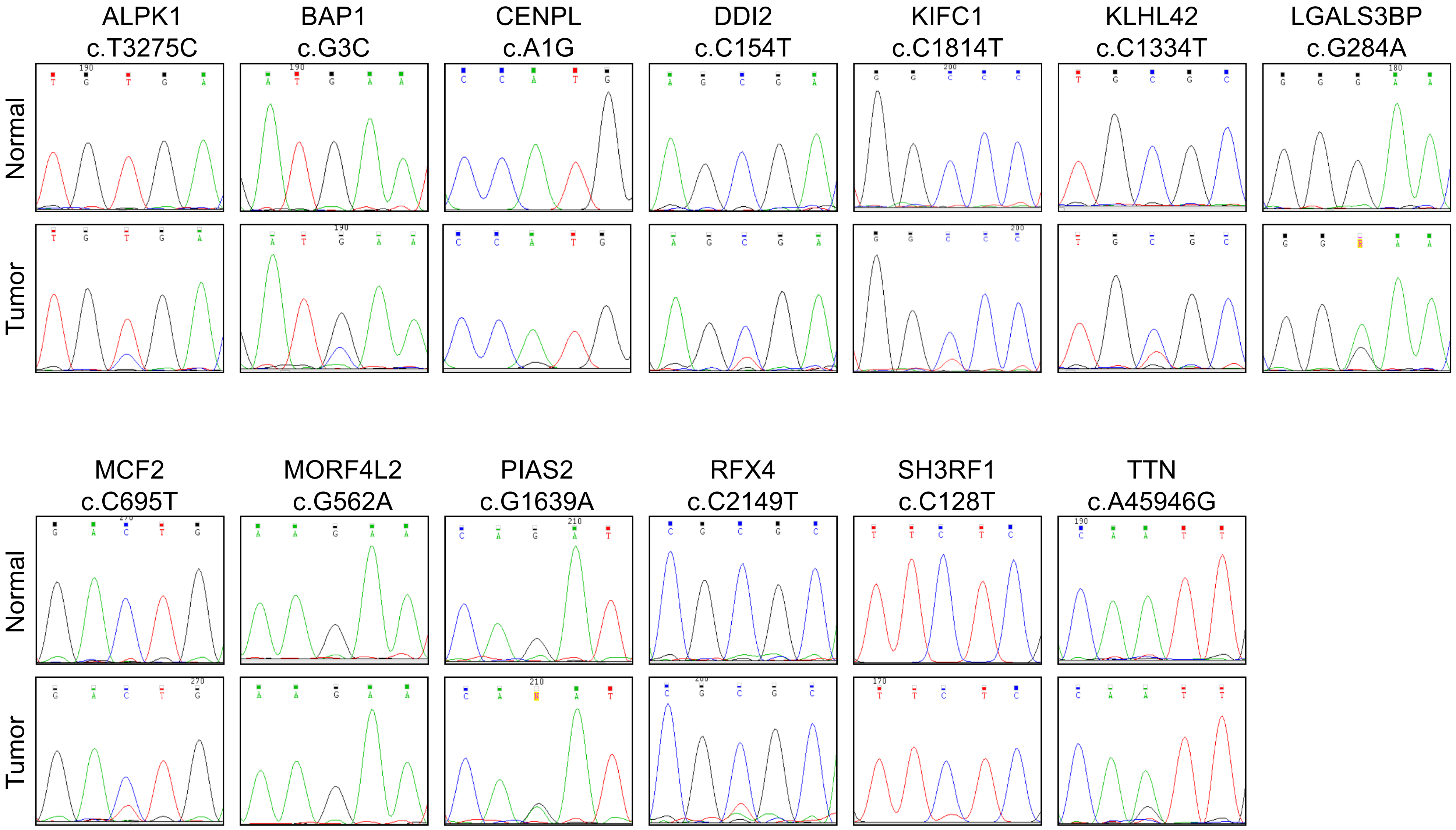
ALPK1
c.T3275C
BAP1
c.G3C
CENPL
c.A1G
DDI2
c.C154T
KIFC1
c.C1814T
KLHL42
c.C1334T
LGALS3BP
c.G284A
Normal
Tumor
MCF2
c.C695T
MORF4L2
c.G562A
PIAS2
c.G1639A
RFX4
c.C2149T
SH3RF1
c.C128T
TTN
c.A45946G
Normal
Tumor

Figure S5

Figure S5: Dideoxy sequencing confirmation of novel SNVs found in a metastatic MPTT.
Sequence tracings from paired normal (top) and tumor (bottom) tissue confirming SNVs found by WGS in a metastatic MPTT. Text above the tracings indicate the gene and coding sequence mutation identified by WGS.

	SNVs	Indels	Mutations
**Total**	10,613	7,507	18,120
**In dbSNP v137**	4,063	2,076	6,139
**Potentially novel**	6,550	5,431	11,981
**Exonic**	37	5	43
**Splicing**	0	0	0
**ncRNA**	218	172	390
**5’ UTR**	12	4	16
**3’ UTR**	52	36	88
**Intronic**	2,080	2,045	4,125
**Intergenic**	4,073	3,098	7,171
**Up/downstream**	73	75	148

Table S1: Summary of mutational calls made by WGS pipeline (VarScan).
Summary of the various mutational (SNVs and indels) calls made by WGS pipeline; specifically, those made by VarScan v2.3.5. The calls made by MuTect v1.1.3 and Strelka v1.0.6 were similar/overlapping and are combined with the VarScan calls in **Tables S2-4**.

Gene	Coding change	Protein change	Functional prediction	COSMIC ID
ADAD2*	chr16.84229293.C>T	R348C	passenger	—
ALPK1*	chr4.113359726.T>C	V1092A	passenger	—
ANKRD20A1	chr9.67934829.G>A	R200Q	driver	—
ANKRD36B	chr12.98173423.T>C	M349T	passenger	—
ASPM	chr1.197069995.G>C	E2796Q	passenger	—
BAP1	chr3.52443892.G>C	M1I	passenger	480297
C2ORF42	chr2.70408595.G>A	E175K	passenger	—
CENPL	chr1.173780437.A>G	M1V	passenger	—
CLCN4*	chrX.10181805.C>T	A554V	passenger	1238524
CST7	chr20.24940348.A>C	*146C	passenger	—
DCAF12L1	chrX.125685226.G>T	G456W	driver	—
DDI1	chr11.103907704.C>T	R52*	passenger	—
EXT2*	chr11.44255774.C>G	T639R	passenger	—
FTH1	chr11.61734852.G>T	D16Y	passenger	—
GLRA1	chr5.151202535.A>G	N358S	passenger	—
IRS2	chr13.110437353.A>C	T350P	passenger	—
KIFC1	chr6.33374250.C>T	A605V	passenger	—
KLHL42	chr12.27950915.C>T	A445V	passenger	—
LAT*	chr16.28998139.A>C	T169P	passenger	—
LGALS3BP	chr17.76970862.G>A	G95E	passenger	—
LUZP1	chr1.23419592.insT	H388fs	passenger	—
MAP1LC3A*	chr20.33147218.C>T	P55L	passenger	—
MCF2*	chrX.138701858.C>T	T232I	passenger	—
MINOS1-NBL1*	chr1.19983540.A>C	H169P	passenger	—
MORF4L2	chrX.102931394.G>A	E188K	passenger	—
NBEAL2	chr3.47039983.G>A	R1050Q	passenger	—
NBPF11*	chr1.146037549.C>G	P718A	passenger	—
NDUFAF7	chr2.37469825.C>A	H224N	passenger	—
NLRP12	chr19.54314294.C>T	P207S	passenger	—
OPN1LW	chrX.153409795.G>A	R13H	passenger	4640949
OR2M4	chr1.248402820.G>C	R197T	passenger	—
PARP4	chr13.25044054.A>G	N675S	passenger	—
PDE1A	chr2.183291317.G>A	T14K	passenger	—
PIAS2	chr18.44400905.G>A	D547N	passenger	—
PIK3CA	chr3.178952085.A>G	H1047R	driver	775
POTEM	chr14.20011648.A>T	T274S	passenger	—
RFX4*	chr12.107155161.C>T	R708C	passenger	1223609
RLN2	chr9.5304107.G>A	G101S	passenger	—
SH3RF1	chr4.170190236.C>T	S43F	passenger	—
TMEM232	chr5.109954179.G>A	V285M	passenger	—
TMPRSS11A*	chr4.68780309.C>A	C367*	passenger	—
TTN*	chr2.179475987.A>G	I15316V	passenger	—
USP17L20	chr4.9269777.C>G	P145A	passenger	—

Table S2: List of somatic mutations (SNVs and indels) found in a metastatic MPTT.
* = gene with multiple transcripts and/or protein isoforms; only the canonical sequence is shown for the protein change. Nucleotide coding changes are based on GRCh37/hg19. Functional protein predictions were made by fathmm[18]. A COSMIC[19] ID number is provided for any previously identified somatic cancer-associated mutation.

Chromosome	Start position	End position	Copy number	CNV type	Genes involved
1	550000	600000	1	LOSS	OR4F16
3	9550000	9700000	3	GAIN	LHFPL4, MTMR14
3	12750000	12850000	3	GAIN	CAND2, TMEM40
3	23550000	23800000	3	GAIN	UBE2E2
3	27500000	27800000	3	GAIN	EOMES, SLC4A7
3	31750000	32050000	3	GAIN	OSBPL10, ZNF860
3	39450000	39750000	3	GAIN	MOBP, RPSA
3	41600000	41750000	1	LOSS	ULK4
3	42550000	43150000	1	LOSS	CCBP2, CCDC13, CYP8B1, FAM198A, GTDC2, HHATL, HIGD1A, KBTBD5, KRBOX1, NKTR, SEC22C, SS18L2, VIPR1, ZBTB47, ZNF662
3	45500000	45800000	1	LOSS	LARS2, LIMD1, SACM1L
3	46900000	47250000	1	LOSS	CCDC12, MYL3, NBEAL2, PTH1R, SETD2
3	47750000	48050000	1	LOSS	DHX30, MAP4, SMARCC1
3	48300000	49800000	1	LOSS	AMIGO3, AMT, APEH, ARIH2, ATRIP, BSN, C3ORF62, C3ORF71, CCDC36, CCDC51, CCDC71, CCDC72, CELSR3, COL7A1, DAG1, DALRD3, FBXW12, GMPPB, GPX1, IMPDH2, IP6K1, IP6K2, KLHDC8B, LAMB2, LOC646498, MST1, NCKIPSD, NDUFAF3, NICN1, NME6, P4HTM, PFKFB4, PLXNB1, PRKAR2A, QARS, QRICH1, RHOA, RNF123, SHISA5, SLC25A20, SLC26A6, SPINK8, TCTA, TMEM89, TREX1, UCN2, UQCRC1, USP19, USP4, WDR6, ZNF589
3	50200000	50700000	1	LOSS	C3ORF18, C3ORF45, CACNA2D2, CISH, CYB561D2, GNAI2, GNAT1, HEMK1, HYAL1, HYAL2, HYAL3, IFRD2, MAPKAPK3, NAT6, NPRL2, RASSF1, SEMA3B, SEMA3F, SLC38A3, TMEM115, TUSC2, ZMYND10
3	51450000	51750000	1	LOSS	GRM2, RAD54L2, TEX264, VPRBP
3	52200000	52900000	1	LOSS	ALAS1, BAP1, C3ORF78, DNAH1, GLT8D1, GLYCTK, GNL3, ITIH1, ITIH3, ITIH4, MUSTN1, NEK4, NISCH, NT5DC2, PBRM1, PHF7, PPM1M, SEMA3G, SPCS1, STAB1, TLR9, TMEM110, TMEM110-MUSTN1, TNNC1, TWF2, WDR82
3	53500000	53950000	1	LOSS	ACTR8, CACNA1D, CHDH, IL17RB, SELK
3	54000000	54150000	1	LOSS	SELK, CACNA2D3
3	57750000	58700000	1	LOSS	ABHD6, ACOX2, DNASE1L3, FAM107A, FAM3D, FLNB, KCTD6, PDHB, PXK, RPP14, SLMAP
3	60900000	61250000	1	LOSS	FHIT
3	61500000	62450000	1	LOSS	C3ORF14, CADPS, FEZF2, PTPRG
3	63850000	64050000	1	LOSS	ATXN7, PSMD6
3	65350000	66550000	1	LOSS	LRIG1, MAGI1, SLC25A26
3	67300000	67750000	1	LOSS	SUCLG2
3	71000000	71500000	1	LOSS	FOXP1
3	72150000	72550000	1	LOSS	RYBP
3	73000000	73150000	1	LOSS	EBLN2, GXYLT2, PPP4R2
3	75400000	75650000	3	GAIN	FAM86DP
3	167150000	167350000	3	GAIN	SERPINI2, WDR49
3	171150000	171300000	3	GAIN	TNIK
3	177750000	178000000	3	GAIN	LINC00578, KCNMB2
3	178150000	178450000	3	GAIN	KCNMB2
3	183600000	183850000	3	GAIN	ABCC5, HTR3C, HTR3D, HTR3E, PARL
3	184100000	184250000	3	GAIN	CHRD
3	184750000	184900000	3	GAIN	C3ORF70, VPS8
3	185400000	185750000	3	GAIN	IGF2BP2, TRA2B
3	187150000	187350000	3	GAIN	RTP4, SST
3	189550000	189850000	3	GAIN	LEPREL1, TP63
3	194500000	194700000	3	GAIN	LOC100507391
6	67250000	67700000	1	LOSS	SLC25A51P1, BAI3
6	67800000	68000000	1	LOSS	SLC25A51P1, BAI3
6	70350000	70550000	1	LOSS	LMBRD1
6	74150000	74300000	1	LOSS	EEF1A1, MB21D1, MTO1
6	75900000	76050000	1	LOSS	COL12A1, COX7A2, FILIP1, TMEM30A
6	80300000	80500000	1	LOSS	SH3BGRL2
6	80800000	81200000	1	LOSS	BCKDHB
6	81950000	82100000	1	LOSS	BCKDHB, FAM46A
6	82400000	82650000	1	LOSS	FAM46A
6	84950000	85200000	1	LOSS	KIAA1009, TBX18
6	86150000	86350000	1	LOSS	NT5E, SNX14, SYNCRIP
6	86750000	87350000	1	LOSS	SNHG5, HTR1E
6	87800000	88000000	1	LOSS	GJB7, ZNF292
6	88200000	88450000	1	LOSS	AKIRIN2, ORC3, RARS2, SLC35A1
6	89600000	89800000	1	LOSS	PNRC1, RNGTT
6	90350000	90550000	1	LOSS	MDN1
6	90650000	91150000	1	LOSS	BACH2
6	92700000	93500000	1	LOSS	MIR4643, EPHA7

Chromosome	Start position	End position	Copy number	CNV type	Genes involved
6	95950000	96050000	1	LOSS	MANEA
6	100900000	101100000	1	LOSS	ASCC3, SIM1
6	106950000	107100000	1	LOSS	AIM1, QRSL1, RTN4IP1
6	107150000	107250000	1	LOSS	LOC100422737
6	108800000	109100000	1	LOSS	FOXO3, LACE1
6	110950000	112050000	1	LOSS	AMD1, CDK19, FYN, GTF3C6, KIAA1919, REV3L, RPF2, SLC16A10, TRAF3IP2
6	116200000	116600000	1	LOSS	COL10A1, FRK, NT5DC1, TSPYL1, TSPYL4
6	119500000	119700000	1	LOSS	MAN1A1
6	122700000	122900000	1	LOSS	HSF2, SERINC1
6	125200000	125700000	1	LOSS	HDDC2, RNF217, TPD52L1
6	126100000	126450000	1	LOSS	HINT3, NCOA7, TRMT11
6	127550000	127700000	1	LOSS	ECHDC1, RNF146
6	128300000	128850000	1	LOSS	PTPRK
6	131100000	131200000	1	LOSS	EPB41L2, LOC100507203
6	131450000	131600000	1	LOSS	AKAP7
6	131850000	132050000	1	LOSS	ARG1, CTAGE9, ENPP3, MED23, OR2A4
6	132150000	132300000	1	LOSS	CTGF, ENPP1
6	132900000	133150000	1	LOSS	RPS12, SLC18B1, TAAR1, TAAR2, TAAR5, VNN1, VNN2
6	135150000	135350000	1	LOSS	ALDH8A1, HBS1L
6	136300000	137150000	1	LOSS	BCLAF1, FAM54A, MAP3K5, MAP7, PDE7B, PEX7
6	137500000	137750000	1	LOSS	IFNGR1
6	138700000	138900000	1	LOSS	HEBP2, NHSL1
6	139250000	139350000	1	LOSS	REPS1
6	140150000	140400000	1	LOSS	LOC100132735
6	142500000	142700000	1	LOSS	GPR126, VTA1
6	143350000	143600000	1	LOSS	AIG1
6	144650000	144800000	1	LOSS	UTRN
6	145950000	146300000	1	LOSS	EPM2A, FBXO30, SHPRH
6	150700000	151100000	1	LOSS	IYD, PLEKHG1
6	154400000	154450000	1	LOSS	OPRM1
6	154700000	154850000	1	LOSS	CNKSR3
6	157100000	157400000	1	LOSS	ARID1B
6	157800000	158050000	1	LOSS	ZDHHC14
6	158250000	158500000	1	LOSS	SNX9, SYNJ2
6	159200000	159250000	1	LOSS	EZR
6	160000000	160250000	1	LOSS	ACAT2, MRPL18, PNLDC1, SOD2, TCP1, WTAP
6	160350000	160600000	1	LOSS	IGF2R, SLC22A1
6	160750000	161200000	1	LOSS	LPA, PLG, SLC22A3
6	163750000	164200000	1	LOSS	QKI
6	166750000	166850000	1	LOSS	BRP44L, RPS6KA2, SFT2D1
6	167400000	167550000	1	LOSS	CCR6, FGFR1OP
6	167650000	168350000	1	LOSS	MLLT4, TCP10, TTLL2, UNC93A
6	168550000	168800000	1	LOSS	DACT2
6	170050000	170350000	1	LOSS	C6ORF120, C6ORF70, PHF10, TCTE3, WDR27
7	80800000	83350000	3	GAIN	CACNA2D1, HGF, PCLO, SEMA3E
7	83350000	83450000	4	GAIN	SEMA3E, SEMA3A
7	83450000	83550000	3	GAIN	SEMA3E, SEMA3A
7	83550000	83950000	4	GAIN	SEMA3A
7	83950000	84700000	3	GAIN	SEMA3D
7	84700000	84900000	4	GAIN	SEMA3D
7	84900000	85000000	3	GAIN	SEMA3D, GRM3
7	85000000	85150000	4	GAIN	SEMA3D, GRM3
7	85150000	86250000	3	GAIN	SEMA3D, GRM3
7	86250000	86750000	4	GAIN	GRM3, KIAA1324L
7	86750000	87450000	3	GAIN	ABCB1, ABCB4, C7ORF23, CROT, DMTF1, RUNDC3B
7	87450000	87800000	4	GAIN	ADAM22, DBF4, RUNDC3B, SLC25A40
7	87800000	88150000	3	GAIN	ADAM22, SRI, STEAP4
7	88300000	88450000	3	GAIN	C7ORF62, ZNF804B
7	88450000	88550000	4	GAIN	ZNF804B
7	88550000	89350000	3	GAIN	ZNF804B
7	89350000	89750000	4	GAIN	DPY19L2P4

Chromosome	Start position	End position	Copy number	CNV type	Genes involved
7	89750000	90500000	3	GAIN	C7ORF63, CDK14, CLDN12, GTPBP10, STEAP1, STEAP2
7	90500000	91600000	4	GAIN	AKAP9, CDK14, FZD1, MTERF
7	91600000	91700000	3	GAIN	AKAP9
7	91700000	92150000	4	GAIN	AKAP9, ANKIB1, CYP51A1, GATAD1, KRIT1, LRRD1, PEX1
7	92150000	92450000	3	GAIN	CDK6, FAM133B, PEX1, RBM48
7	92450000	96450000	4	GAIN	ASB4, BET1, CALCR, CASD1, CCDC132, CDK6, COL1A2, DYNC1I1, FLJ42280, GNG11, GNGT1, HEPACAM2, PDK4, PEG10, PON1, PON2, PON3, PPP1R9A, SAMD9, SAMD9L, SGCE, SHFM1, SLC25A13, TFPI2
7	96450000	100150000	3	GAIN	ACN9, AGFG2, AP4M1, ARPC1A, ARPC1B, ASNS, ATP5J2, ATP5J2-PTCD1, AZGP1, BAIAP2L1, BHLHA15, BRI3, BUD31, C7ORF43, C7ORF59, C7ORF61, CNPY4, COPS6, CPSF4, CYP3A4, CYP3A43, CYP3A5, CYP3A7, CYP3A7-CYP3AP1, DLX5, DLX6, FAM200A, GAL3ST4, GATS, GJC3, GPC2, KPNA7, LMTK2, LOC100289187, MBLAC1, MCM7, MEPCE, NPTX2, NYAP1, OCM2, OR2AE1, PDAP1, PILRA, PILRB, PPP1R35, PTCD1, PVRIG, SMURF1, SPDYE3, STAG3, TAC1, TAF6, TECPR1, TMEM130, TRIM4, TRRAP, TSC22D4, ZCWPW1, ZKSCAN1, ZKSCAN5, ZNF3, ZNF394, ZNF498, ZNF655, ZNF789, ZSCAN21
8	300000	500000	1	LOSS	C8ORF42, FBXO25
8	1650000	2050000	1	LOSS	ARHGEF10, CLN8, KBTBD11, MYOM2
8	2700000	3400000	1	LOSS	CSMD1
8	6800000	7950000	1	LOSS	DEFA1, DEFA1B, DEFA3, DEFA5, DEFB103A, DEFB103B, DEFB104A, DEFB104B, DEFB105A, DEFB105B, DEFB106A, DEFB106B, DEFB107A, DEFB107B, DEFB4A, DEFB4B, LOC100132396, SPAG11A, SPAG11B, USP17L1P, USP17L3, USP17L4, USP17L8, ZNF705G
8	8550000	9500000	1	LOSS	CLDN23, ERI1, MFHAS1, PPP1R3B, TNKS
8	10550000	10700000	1	LOSS	C8ORF74, PINX1, SOX7
8	11300000	12100000	1	LOSS	BLK, CTSB, DEFB130, DEFB134, DEFB135, DEFB136, FAM167A, FAM86B1, FDFT1, GATA4, LOC100133267, NEIL2, USP17L2, USP17L7, ZNF705D
8	12200000	12400000	1	LOSS	FAM86B2
8	12800000	14300000	1	LOSS	C8ORF48, DLC1, KIAA1456, SGCZ
8	87600000	91000000	3	GAIN	CNBD1, CNGB3, DCAF4L2, MMP16, NBN, OSGIN2, RIPK2
8	91100000	97500000	3	GAIN	C8ORF37, C8ORF87, CCNE2, CDH17, DPY19L4, ESRP1, FAM92A1, FSBP, GDF6, GEM, INTS8, KIAA1429, LOC100127983, LRRC69, MTERFD1, NDUFAF6, NECAB1, OTUD6B, PDP1, PLEKHF2, PTDSS1, RAD54B, RBM12B, RUNX1T1, SLC26A7, TMEM55A, TMEM64, TMEM67, TP53INP1, TRIQK, UQCRB
8	97900000	98100000	3	GAIN	CPQ
8	98350000	101450000	3	GAIN	C8ORF47, COX6C, FBXO43, HRSP12, KCNS2, LAPTM4B, MATN2, MTDH, NIPAL2, OSR2, POLR2K, POP1, RGS22, RNF19A, RPL30, SPAG1, STK3, VPS13B
8	101500000	103800000	3	GAIN	ANKRD46, GRHL2, KLF10, NCALD, ODF1, PABPC1, RRM2B, SNX31, UBR5, YWHAZ, ZNF706
8	103950000	105250000	3	GAIN	ATP6V1C1, BAALC, CTHRC1, DCAF13, FZD6, RIMS2, SLC25A32
8	105500000	107600000	3	GAIN	LRP12, OXR1, ZFPM2
8	107750000	110550000	3	GAIN	ABRA, ANGPT1, EIF3E, EMC2, ENY2, NUDCD1, OXR1, PKHD1L1, RSPO2, TMEM74, TRHR
8	110650000	118800000	3	GAIN	AARD, CSMD3, EIF3H, KCNV1, MED30, RAD21, SLC30A8, SYBU, TRPS1, UTP23
8	119150000	121650000	3	GAIN	COL14A1, COLEC10, DEPTOR, DSCC1, ENPP2, MAL2, MRPL13, MTBP, NOV, SAMD12, SNTB1, TAF2, TNFRSF11B
8	121900000	122950000	3	GAIN	HAS2
8	123050000	123850000	3	GAIN	ZHX2
8	124050000	125550000	3	GAIN	ANXA13, ATAD2, C8ORF76, DERL1, FAM83A, FAM91A1, FBXO32, FER1L6, KLHL38, RNF139, TATDN1, TMEM65, TRMT12, WDR67, WDYHV1, ZHX1, ZHX1-C8ORF76
8	125800000	126400000	3	GAIN	KIAA0196, NSMCE2, SQLE, ZNF572
8	126600000	129500000	3	GAIN	FAM84B, MYC, POU5F1B
8	129600000	132850000	3	GAIN	ADCY8, ASAP1, FAM49B, GSDMC
8	133100000	134250000	3	GAIN	HHLA1, KCNQ3, LRRC6, PHF20L1, SLA, TG, TMEM71, WISP1
8	134600000	135700000	3	GAIN	ZFAT
8	135850000	136450000	3	GAIN	LOC286094
8	136600000	137600000	3	GAIN	KHDRBS3
8	137900000	140900000	3	GAIN	COL22A1, FAM135B, KCNK9, TRAPPC9
8	141000000	144600000	3	GAIN	ARC, BAI1, C8ORF31, CHRAC1, CYP11B1, CYP11B2, DENND3, EIF2C2, FLJ43860, GLI4, GML, GPIHBP1, GPR20, JRK, LY6D, LY6E, LY6H, LY6K, LYNX1, LYPD2, MAFA, PSCA, PTK2, PTP4A3, RHPN1, SLC45A4, SLURP1, THEM6, TOP1MT, TRAPPC9, TSNARE1, ZC3H3, ZFP41, ZNF696
8	144750000	144850000	3	GAIN	CCDC166, FAM83H, MAPK15, ZNF707
8	145050000	145100000	3	GAIN	GRINA, PARP10, SPATC1
8	145200000	145300000	3	GAIN	HEATR7A
8	145750000	145950000	3	GAIN	ARHGAP39, C8ORF82, LRRC24, ZNF251
8	146100000	146400000	3	GAIN	C8ORF33, ZNF16, ZNF250
12	700000	1050000	1	LOSS	NINJ2, RAD52, WNK1
12	1750000	2150000	1	LOSS	ADIPOR2, CACNA2D4, DCP1B, LRTM2, WNT5B
12	3200000	3450000	1	LOSS	TSPAN9
12	3900000	4150000	1	LOSS	PARP11
12	6250000	6700000	1	LOSS	CD27, CD9, CHD4, GAPDH, IFFO1, LTBR, MRPL51, NCAPD2, NOP2, PLEKHG6, SCNN1A, TAPBPL, TNFRSF1A, VAMP1
12	6850000	7350000	1	LOSS	ATN1, C12ORF57, C1R, C1RL, C1S, CD4, CDCA3, CLSTN3, EMG1, ENO2, GNB3, GPR162, LAG3, LEPREL2, LPCAT3, LRRC23, MLF2, PEX5, PHB2, PTMS, PTPN6, RBP5, SPSB2, TPI1, USP5
12	9200000	9500000	1	LOSS	A2M, PZP
12	11000000	11250000	1	LOSS	PRH1, PRH2, PRR4, TAS2R13, TAS2R14, TAS2R19, TAS2R20, TAS2R31, TAS2R43, TAS2R46, TAS2R50
12	11750000	12050000	1	LOSS	ETV6
12	12250000	12800000	1	LOSS	BCL2L14, CREBL2, DUSP16, LOH12CR1, LRP6, MANSC1
12	15850000	16650000	1	LOSS	DERA, MGST1, SLC15A5, STRAP

Chromosome	Start position	End position	Copy number	CNV type	Genes involved
12	17100000	18800000	1	LOSS	PIK3C2G, RERGL
12	19250000	19500000	1	LOSS	PLEKHA5
12	20850000	22100000	1	LOSS	ABCC9, C12ORF39, GOLT1B, GYS2, IAPP, KCNJ8, LDHB, PYROXD1, RECQL, SLCO1A2, SLCO1B1, SLCO1B3, SLCO1B7, SLCO1C1
12	22550000	22850000	1	LOSS	ETNK1, KIAA0528
12	23650000	24750000	1	LOSS	SOX5
12	26450000	27550000	1	LOSS	ARNTL2, ASUN, C12ORF71, FGFR1OP2, ITPR2, MED21, STK38L, TM7SF3
12	28300000	28750000	1	LOSS	CCDC91
12	29450000	29750000	1	LOSS	ERGIC2, FAR2, OVCH1, TMTC1
12	51000000	51050000	3	GAIN	DIP2B
12	51050000	51950000	4	GAIN	ATF1, BIN2, CELA1, CSRNP2, DAZAP2, DIP2B, GALNT6, HIGD1C, LETMD1, METTL7A, POU6F1, SLC11A2, SLC4A8, SMAGP, TFCP2, TMPRSS12
12	51950000	52000000	3	GAIN	SCN8A
12	69450000	69500000	3	GAIN	CPM, CPSF6
12	69500000	71950000	4	GAIN	BEST3, CCT2, CNOT2, CPSF6, FRS2, KCNMB4, LGR5, LRRC10, LYZ, PTPRB, PTPRR, RAB3IP, TSPAN8, YEATS4
12	71950000	72000000	3	GAIN	LGR5
13	1	115200000	3	GAIN	A2LD1, ABCC4, ABHD13, ADPRHL1, AKAP11, ALG11, ALG5, ALOX5AP, ANKRD10, ARGLU1, ARHGEF7, ARL11, ATP11A, ATP12A, ATP4B, ATP7B, ATP8A2, B3GALTL, BIVM, BIVM-ERCC5, BORA, BRCA2, C13ORF33, C13ORF35, C1QTNF9, C1QTNF9B, C1QTNF9B-AS1, CAB39L, CARKD, CARS2, CCDC122, CCDC168, CCDC169, CCDC169-SOHLH2, CCDC70, CCNA1, CDADC1, CDC16, CDK8, CDX2, CENPJ, CHAMP1, CKAP2, CLDN10, CLN5, CLYBL, COG3, COG6, COL4A1, COL4A2, COMMD6, CPB2, CRYL1, CSNK1A1L, CUL4A, CYSLTR2, DACH1, DAOA, DCLK1, DCT, DCUN1D2, DGKH, DHRS12, DIAPH3, DIS3, DLEU7, DNAJC15, DNAJC3, DOCK9, DZIP1, EBPL, EDNRB, EFHA1, EFNB2, ELF1, ENOX1, EPSTI1, ERCC5, ESD, EXOSC8, F10, F7, FAM123A, FAM124A, FAM155A, FAM194B, FAM216B, FAM48A, FAM70B, FARP1, FBXL3, FGF14, FGF9, FLT1, FLT3, FNDC3A, FOXO1, FREM2, FRY, GAS6, GJA3, GJB2, GJB6, GPC5, GPC6, GPR12, GPR18, GPR180, GPR183, GRK1, GRTP1, GSX1, GTF2F2, GTF3A, HMGB1, HNRNPA1L2, HS6ST3, HSPH1, HTR2A, IFT88, IL17D, ING1, INTS6, IPO5, IRG1, IRS2, ITGBL1, ITM2B, KATNAL1, KBTBD6, KBTBD7, KCNRG, KCTD12, KCTD4, KDELC1, KIAA0226L, KIAA0564, KIAA1704, KL, KLF12, KLF5, KLHL1, KPNA3, LACC1, LAMP1, LATS2, LCP1, LECT1, LHFP, LIG4, LINC00364, LMO7, LNX2, LOC729420, LPAR6, LRCH1, MAB21L1, MBNL2, MCF2L, MED4, METTL21C, MIPEP, MLNR, MPHOSPH8, MRP63, MRPS31, MTIF3, MTMR6, MTRF1, MTUS2, MYCBP2, MYO16, MZT1, N4BP2L1, N4BP2L2, N6AMT2, NAA16, NALCN, NBEA, NDFIP2, NEK3, NEK5, NHLRC3, NUDT15, NUFIP1, NUPL1, OLFM4, OXGR1, PABPC3, PAN3, PARP4, PCCA, PCDH17, PCDH20, PCDH8, PCDH9, PCID2, PDS5B, PDX1, PHF11, PIBF1, POLR1D, POMP, POSTN, POU4F1, PRHOXNB, PROSER1, PROZ, PRR20A, PRR20B, PRR20C, PRR20D, PRR20E, PSPC1, RAB20, RAP2A, RASA3, RASL11A, RB1, RBM26, RCBTB1, RCBTB2, RFC3, RFXAP, RGCC, RNASEH2B, RNF113B, RNF17, RNF219, RNF6, RPL21, RXFP2, SACS, SAP18, SCEL, SERP2, SERPINE3, SERTM1, SETDB2, SGCG, SHISA2, SIAH3, SKA3, SLAIN1, SLC10A2, SLC15A1, SLC25A15, SLC25A30, SLC46A3, SLC7A1, SLITRK1, SLITRK5, SLITRK6, SMAD9, SOHLH2, SOX1, SOX21, SPACA7, SPATA13, SPERT, SPG20, SPRY2, SPRYD7, STARD13, STK24, STOML3, SUCLA2, SUGT1, TBC1D4, TDRD3, TEX26, TEX29, TEX30, TFDP1, TGDS, THSD1, TM9SF2, TMCO3, TMTC4, TNFRSF19, TNFSF11, TNFSF13B, TPP2, TPT1, TPTE2, TRIM13, TRPC4, TSC22D1, TUBA3C, TUBGCP3, UBAC2, UBL3, UCHL3, UFM1, UGGT2, UPF3A, USP12, USPL1, UTP14C, VPS36, WASF3, WBP4, WDFY2, XPO4, ZAR1L, ZC3H13, ZDHHC20, ZIC2, ZIC5, ZMYM2, ZMYM5
14	92200000	94250000	3	GAIN	ATXN3, BTBD7, C14ORF109, C14ORF142, CHGA, COX8C, CPSF2, FBLN5, GOLGA5, ITPK1, LGMN, MOAP1, NDUFB1, PRIMA1, RIN3, SLC24A4, TC2N, TRIP11, UBR7, UNC79
14	97700000	103400000	3	GAIN	AMN, ANKRD9, BCL11B, BEGAIN, C14ORF177, CCDC85C, CCNK, CINP, CYP46A1, DEGS2, DIO3, DLK1, DYNC1H1, EML1, EVL, HHIPL1, HSP90AA1, MOK, PPP2R5C, RCOR1, RTL1, SETD3, SLC25A29, SLC25A47, TECPR2, TRAF3, WARS, WDR20, WDR25, YY1, ZNF839
14	103600000	104000000	3	GAIN	CKB, EIF5, MARK3, TNFAIP2, TRMT61A
14	104800000	106100000	3	GAIN	ADSSL1, AHNAK2, AKT1, BRF1, BTBD6, C14ORF180, C14ORF79, C14ORF80, CDCA4, CRIP1, CRIP2, GPR132, INF2, JAG2, KIAA0284, MTA1, NUDT14, PACS2, PLD4, SIVA1, TEX22, TMEM121, TMEM179, ZBTB42
14	106100000	106150000	4	GAIN	ELK2AP
14	106150000	106500000	3	GAIN	ADAM6, KIAA0125
14	106500000	106550000	4	GAIN	ADAM6, LINC00226
14	106550000	107100000	3	GAIN	LINC00221, LINC00226
16	68600000	68750000	3	GAIN	CDH3
17	1200000	2350000	1	LOSS	CRK, DPH1, HIC1, INPP5K, METTL16, MNT, MYO1C, OVCA2, PITPNA, PRPF8, RILP, RPA1, RTN4RL1, SCARF1, SERPINF1, SERPINF2, SGSM2, SLC43A2, SMG6, SMYD4, SRR, TLCD2, TSR1, TUSC5, WDR81, YWHAE
17	3700000	4250000	1	LOSS	ANKFY1, ATP2A3, C17ORF85, CAMKK1, CYB5D2, ITGAE, P2RX1, UBE2G1, ZZEF1
17	4500000	5400000	1	LOSS	ALOX15, ARRB2, C17ORF107, C1QBP, CAMTA2, CHRNE, CXCL16, DERL2, DHX33, ENO3, GLTPD2, GP1BA, INCA1, KIF1C, MED11, MINK1, MIS12, NUP88, PELP1, PFN1, PLD2, PSMB6, RABEP1, RNF167, RPAIN, SCIMP, SLC25A11, SLC52A1, SMTNL2, SPAG7, TM4SF5, USP6, VMO1, ZFP3, ZMYND15, ZNF232, ZNF594
17	6550000	7550000	1	LOSS	ACADVL, ACAP1, ALOX12, ASGR1, ASGR2, BCL6B, C17ORF100, C17ORF49, C17ORF61, C17ORF74, C17ORF81, CD68, CHRNB1, CLDN7, CLEC10A, CTDNEP1, DLG4, DVL2, EIF4A1, EIF5A, FBXO39, FGF11, FXR2, GABARAP, GPS2, KCTD11, MED31, MPDU1, NEURL4, NLGN2, PHF23, PLSCR3, POLR2A, RNASEK, SAT2, SENP3, SHBG, SLC13A5, SLC16A11, SLC16A13, SLC2A4, SLC35G6, SOX15, SPEM1, TEKT1, TMEM102, TMEM95, TNFSF12, TNFSF12-TNFSF13, TNFSF13, TNK1, XAF1, YBX2, ZBTB4
17	8000000	8550000	1	LOSS	ALOXE3, ARHGEF15, AURKB, C17ORF59, CTC1, HES7, KRBA2, MYH10, NDEL1, ODF4, PER1, PFAS, RANGRF, RNF222, RPL26, SLC25A35, TMEM107, VAMP2
17	8900000	9150000	1	LOSS	NTN1
17	10450000	10800000	1	LOSS	C17ORF48, MYH2, MYH3, PIRT, SCO1, TMEM220
17	13800000	14350000	1	LOSS	CDRT15, COX10, HS3ST3B1
17	75950000	76150000	7	GAIN	C17ORF99, TMC6, TMC8, TNRC6C
17	76150000	76250000	6	GAIN	AFMID, BIRC5, C17ORF99, SYNGR2, TK1, TMEM235
17	76250000	76300000	5	GAIN	TMEM235, SOCS3
17	76300000	76350000	6	GAIN	TMEM235, SOCS3
17	76350000	76400000	7	GAIN	PGS1, SOCS3
17	76400000	76500000	8	GAIN	DNAH17, PGS1
17	76500000	76950000	5	GAIN	CYTH1, DNAH17, LOC100653515, TIMP2, USP36
17	76950000	77000000	3	GAIN	CANT1, LGALS3BP
17	77150000	77200000	3	GAIN	RBFOX3
17	77200000	77300000	5	GAIN	RBFOX3
17	77300000	77550000	3	GAIN	RBFOX3
17	77600000	77950000	4	GAIN	CBX2, CBX4, CBX8, ENPP7, TBC1D16

Chromosome	Start position	End position	Copy number	CNV type	Genes involved
17	77950000	78150000	3	GAIN	CCDC40, EIF4A3, GAA, TBC1D16
17	80850000	80950000	3	GAIN	B3GNTL1, TBCD
20	550000	800000	3	GAIN	SCRT2, SLC52A3, SRXN1, TCF15
20	1550000	1700000	3	GAIN	SIRPB1, SIRPG
20	5600000	5900000	3	GAIN	C20ORF196, CHGB
20	17300000	17500000	3	GAIN	BFSP1, PCSK2
20	18600000	18750000	3	GAIN	DTD1
21	1	16700000	4	GAIN	BAGE, BAGE2, BAGE3, BAGE4, BAGE5, HSPA13, LIPI, LOC388813, NRIP1, POTED, RBM11, SAMSN1, TPTE
21	16700000	16750000	3	GAIN	NRIP1, USP25
21	17350000	17500000	3	GAIN	LINC00478
21	33400000	33650000	3	GAIN	MIS18A
21	35350000	35450000	3	GAIN	MRPS6
21	35950000	36100000	3	GAIN	CLIC6, RCAN1
21	36600000	36750000	3	GAIN	RUNX1, MIR802
21	40950000	41150000	3	GAIN	B3GALT5, IGSF5
21	46050000	46150000	3	GAIN	KRTAP10-10, KRTAP10-11, KRTAP10-12, KRTAP12-1, KRTAP12-2, KRTAP12-3, KRTAP12-4, TSPEAR
21	47300000	47350000	3	GAIN	PCBP3
21	48000000	48150000	4	GAIN	PRMT2, S100B

Table S3: List of copy number variations (CNVs) found in a metastatic MPTT.
Location of CNVs are based on GRCh37/hg19. Only genes with a copy number gain (> 2) or loss (< 2) are shown.

SV	Breakpoint A					Breakpoint B				
	Type	Chromosome	Position	Genes involved	Orientation	Type	Chromosome	Position	Genes involved	Orientation
CTX	intergenic	1	11000047	CASZ1, C1orf127	+	intergenic	10	130354137	MKI67, MGMT	+
INS	intergenic	1	225939134	ENAH, SRP9	+	intronic	1	225781779	ENAH	+
DEL	exonic	1	247641141	OR2W5	+		1	247676353		+
DEL	intergenic	2	21774847	APOB, LOC645949	+		2	21835934		+
ITX	intronic	2	175712899	CHN1	+	intronic	2	175712989	CHN1	-
CTX	intergenic	3	152826918	P2RY1, RAP2B	+	intronic	10	98189301	TLL2	-
ITX	intergenic	4	133346347	NONE, PCDH10	+	intergenic	4	133346557	NONE, PCDH10	-
CTX	intronic	4	108637397	PAPSS1	+	intronic	3	13664751	FBLN2	+
INS	intergenic	4	40708287	RBM47, NSUN7	+	intronic	4	40541179	RBM47	+
CTX	intergenic	5	160377908	LOC285629, GABRB2	-	intergenic	X	146714796	MIR514A3, FMR1-AS1	+
INS	intronic	6	65730578	EYS	+	intronic	6	62605439	KHDRBS2	+
ITX	intronic	6	62802430	KHDRBS2	+	intronic	6	62801956	KHDRBS2	-
CTX	intergenic	6	168150495	TCP10, C6orf123	+	intronic	3	31765039	OSBPL10	-
ITX	intergenic	7	83325065	SEMA3E, SEMA3A	-	intergenic	7	83324360	SEMA3E, SEMA3A	+
ITX	intergenic	7	96459480	SHFM1, DLX6-AS1	+	intergenic	7	83418038	SEMA3E, SEMA3A	-
DEL	exonic	9	21469738	CDKN2A, CDKN2B, IFNE, MTAP	+		9	22017561		+
DEL	intergenic	9	7681685	KDM4C, C9orf123	+		9	7684556		+
INS	intergenic	9	92846399	LOC286370, LOC340515	+	intergenic	9	92837788	LOC286370, LOC340515	+
INS	intergenic	9	138192365	LOC401557, C9orf62	+	intergenic	9	138037872	OLFM1, LOC401557	+
DEL	exonic	10	131704409	EBF3, GLRX3	+		10	132316339		+
DEL	exonic	12	38372976	AAAS, ABCD2, ACVR1B, ACVRL1, ADAMTS20, ADCY6, AGAP2, ALG10B, AMHR2, AMIGO2, ANKRD33, ANKRD52, ANO6, ANP32D, APOF, AQP2, AQP5, AQP6, ARF3, ARHGAP9, ARHGEF25, ARID2, ASB8, ASIC1, ATF1, ATF7, ATP5B, ATP5G2, AVIL, AVPR1A, B4GALNT1, BAZ2A, BCDIN3D, BEST3, BIN2, BLOC1S1, C12orf10, C12orf40, C12orf44, C12orf54, C12orf56, C12orf61, C12orf66, C12orf68, C1QL4, CACNB3, CALCOCO1, CAND1, CBX5, CCDC65, CCNT1, CCT2, CD63, CDK2, CDK4, CELA1, CERS5, CNOT2, CNPY2, CNTN1, COL2A1, COPZ1, COQ10A, COX14, CPM, CPNE8, CPSF6, CS, CSAD, CSRNP2, CTDSP2, CYP27B1, DAZAP2, DBX2, DCD, DCTN2, DDIT3, DDN, DDX23, DGKA, DHH, DIP2B, DNAJC14, DNAJC22, DPY19L2, DTX3, DYRK2, EIF4B, ENDOU, ERBB3, ESPL1, ESYT1, FAIM2, FAM113B, FAM186A, FAM186B, FAM19A2, FIGNL2, FKBP11, FMNL3, FRS2, GALNT6, GDF11, GLI1, GLS2, GNS, GPD1, GPR182, GPR84, GRASP, GRIP1, GTSF1, GXYLT1, H1FNT, HDAC7, HELB, HIGD1C, HMGA2, HNRNPA1, HOXC10, HOXC11, HOXC12, HOXC13, HOXC4, HOXC5, HOXC6, HOXC8, HOXC9, HSD17B6, IFNG, IGFBP6, IKZF4, IL22, IL23A, IL26, INHBC, INHBE, IRAK3, IRAK4, ITGA5, ITGA7, ITGB7, KANSL2, KCNH3, KCNMB4, KIF21A, KIF5A, KRT1, KRT18, KRT2, KRT3, KRT4, KRT5, KRT6A, KRT6B, KRT6C, KRT7, KRT71, KRT72, KRT73, KRT74, KRT75, KRT76, KRT77, KRT78, KRT79, KRT8, KRT80, KRT81, KRT82, KRT83, KRT84, KRT85, KRT86, LACRT, LALBA, LARP4, LEMD3, LETMD1, LGR5, LIMA1, LLPH, LMBR1L, LOC283403, LRIG3, LRP1, LRRC10, LRRK2, LYZ, MAP3K12, MARCH9, MARS, MBD6, MCRS1, MDM1, MDM2, METTL1, METTL21B, METTL7A, METTL7B, MFSD5, MIP, MLL2, MMP19, MON2, MSRB3, MUCL1, MYL6, MYL6B, MYO1A, NAB2, NACA, NCKAP1L, NCKAP5L, NDUFA4L2, NELL2, NEUROD4, NFE2, NPFF, NR4A1, NUP107, NXPH4, OBFC2B, OR10A7, OR10AD1, OR10P1, OR2AP1, OR6C1, OR6C2, OR6C3, OR6C4, OR6C6, OR6C65, OR6C68, OR6C70, OR6C74, OR6C75, OR6C76, OR8S1, OR9K2, ORMDL2, OS9, PA2G4, PAN2, PCBP2, PDE1B, PDZRN4, PFDN5, PFKM, PIP4K2C, PMEL, POU6F1, PPHLN1, PPM1H, PPP1R1A, PRICKLE1, PRIM1, PRKAG1, PRPF40B, PRPH, PRR13, PTGES3, PTPRB, PTPRR, PUS7L, R3HDM2, RAB3IP, RAB5B, RACGAP1, RAP1B, RAPGEF3, RARG, RASSF3, RBMS2, RDH16, RDH5, RHEBL1, RND1, RNF41, RPAP3, RPL41, RPS26, SARNP, SCAF11, SCN8A, SDR9C7, SENP1, SHMT2, SLC11A2, SLC16A7, SLC26A10, SLC2A13, SLC35E3, SLC38A1, SLC38A2, SLC38A4, SLC39A5, SLC48A1, SLC4A8, SMAGP, SMARCC2, SMARCD1, SMUG1, SOAT2, SP1, SP7, SPATS2, SPRYD3, SPRYD4, SRGAP1, STAC3, STAT2, STAT6, SUOX, TAC3, TARBP2, TBC1D30, TBK1, TENC1, TESPA1, TFCP2, TIMELESS, TMBIM4, TMBIM6, TMEM106C, TMEM117, TMEM194A, TMEM5, TMPRSS12, TROAP, TSFM, TSPAN31, TSPAN8, TUBA1A, TUBA1B, TUBA1C, TWF1, USP15, VDR, WIBG, WIF1, WNT1, WNT10B, XPOT, XRCC6BP1, YAF2, YEATS4, ZBTB39, ZC3H10, ZCRB1, ZNF385A, ZNF641, ZNF740	+		12	71934150		+

	Breakpoint A					Breakpoint B				
SV	Type	Chromosome	Position	Genes involved	Orientation	Type	Chromosome	Position	Genes involved	Orientation
DEL	exonic	12	41918572	AAAS, ACVR1B, ACVRL1, ADAMTS20, ADCY6, AGAP2, AMHR2, AMIGO2, ANKRD33, ANKRD52, ANO6, ANP32D, APOF, AQP2, AQP5, AQP6, ARF3, ARHGAP9, ARHGEF25, ARID2, ASB8, ASIC1, ATF1, ATF7, ATP5B, ATP5G2, AVIL, AVPR1A, B4GALNT1, BAZ2A, BCDIN3D, BIN2, BLOC1S1, C12orf10, C12orf44, C12orf54, C12orf56, C12orf61, C12orf66, C12orf68, C1QL4, CACNB3, CALCOCO1, CAND1, CBX5, CCDC65, CCNT1, CD63, CDK2, CDK4, CELA1, CERS5, CNPY2, COL2A1, COPZ1, COQ10A, COX14, CPM, CS, CSAD, CSRNP2, CTDSP2, CYP27B1, DAZAP2, DBX2, DCD, DCTN2, DDIT3, DDN, DDX23, DGKA, DHH, DIP2B, DNAJC14, DNAJC22, DPY19L2, DTX3, DYRK2, EIF4B, ENDOU, ERBB3, ESPL1, ESYT1, FAIM2, FAM113B, FAM186A, FAM186B, FAM19A2, FIGNL2, FKBP11, FMNL3, GALNT6, GDF11, GLI1, GLS2, GNS, GPD1, GPR182, GPR84, GRASP, GRIP1, GTSF1, GXYLT1, H1FNT, HDAC7, HELB, HIGD1C, HMGA2, HNRNPA1, HOXC10, HOXC11, HOXC12, HOXC13, HOXC4, HOXC5, HOXC6, HOXC8, HOXC9, HSD17B6, IFNG, IGFBP6, IKZF4, IL22, IL23A, IL26, INHBC, INHBE, IRAK3, IRAK4, ITGA5, ITGA7, ITGB7, KANSL2, KCNH3, KIF5A, KRT1, KRT18, KRT2, KRT3, KRT4, KRT5, KRT6A, KRT6B, KRT6C, KRT7, KRT71, KRT72, KRT73, KRT74, KRT75, KRT76, KRT77, KRT78, KRT79, KRT8, KRT80, KRT81, KRT82, KRT83, KRT84, KRT85, KRT86, LACRT, LALBA, LARP4, LEMD3, LETMD1, LIMA1, LLPH, LMBR1L, LOC283403, LRIG3, LRP1, MAP3K12, MARCH9, MARS, MBD6, MCRS1, MDM1, MDM2, METTL1, METTL21B, METTL7A, METTL7B, MFSD5, MIP, MLL2, MMP19, MON2, MSRB3, MUCL1, MYL6, MYL6B, MYO1A, NAB2, NACA, NCKAP1L, NCKAP5L, NDUFA4L2, NELL2, NEUROD4, NFE2, NPFF, NR4A1, NUP107, NXPH4, OBFC2B, OR10A7, OR10AD1, OR10P1, OR2AP1, OR6C1, OR6C2, OR6C3, OR6C4, OR6C6, OR6C65, OR6C68, OR6C70, OR6C74, OR6C75, OR6C76, OR8S1, OR9K2, ORMDL2, OS9, PA2G4, PAN2, PCBP2, PDE1B, PDZRN4, PFDN5, PFKM, PIP4K2C, PMEL, POU6F1, PPHLN1, PPM1H, PPP1R1A, PRICKLE1, PRIM1, PRKAG1, PRPF40B, PRPH, PRR13, PTGES3, PUS7L, R3HDM2, RAB5B, RACGAP1, RAP1B, RAPGEF3, RARG, RASSF3, RBMS2, RDH16, RDH5, RHEBL1, RND1, RNF41, RPAP3, RPL41, RPS26, SARNP, SCAF11, SCN8A, SDR9C7, SENP1, SHMT2, SLC11A2, SLC16A7, SLC26A10, SLC35E3, SLC38A1, SLC38A2, SLC38A4, SLC39A5, SLC48A1, SLC4A8, SMAGP, SMARCC2, SMARCD1, SMUG1, SOAT2, SP1, SP7, SPATS2, SPRYD3, SPRYD4, SRGAP1, STAC3, STAT2, STAT6, SUOX, TAC3, TARBP2, TBC1D30, TBK1, TENC1, TESPA1, TFCP2, TIMELESS, TMBIM4, TMBIM6, TMEM106C, TMEM117, TMEM194A, TMEM5, TMPRSS12, TROAP, TSFM, TSPAN31, TUBA1A, TUBA1B, TUBA1C, TWF1, USP15, VDR, WIBG, WIF1, WNT1, WNT10B, XPOT, XRCC6BP1, YAF2, ZBTB39, ZC3H10, ZCRB1, ZNF385A, ZNF641, ZNF740	+		12	69485203		+
INS	intergenic	12	40959766	LRRK2, CNTN1	+	intergenic	12	40945616	LRRK2, CNTN1	+
CTX	intronic	12	86204701	RASSF9	+	intronic	20	15934827	MACROD2	-
ITX	upstream	12	51984535	SCN8A	+	intergenic	12	39403299	CPNE8, KIF21A	-
DEL	exonic	14	56996042	ACTR10, AKAP5, AP5M1, ARID4A, C14orf101, C14orf105, C14orf135, C14orf149, C14orf37, C14orf38, C14orf39, CHURC1, CHURC1-FNTB, DAAM1, DACT1, DHRS7, ESR2, EXOC5, FAM71D, FNTB, FUT8, GPHB5, GPHN, GPR135, GPX2, HIF1A, HSPA2, JKAMP, KCNH5, KIAA0586, MAX, MNAT1, MPP5, MTHFD1, NAA30, OTX2, PLEKHG3, PPM1A, PPP1R36, PPP2R5E, PRKCH, PSMA3, RAB15, RHOJ, RTN1, SGPP1, SIX1, SIX4, SIX6, SLC35F4, SLC38A6, SNAPC1, SPTB, SYNE2, SYT16, TIMM9, TMEM30B, TOMM20L, TRMT5, WDR89, ZBTB1, ZBTB25	+		14	67783283		+
INS	intergenic	14	106574471	ADAM6, LINC00226	+	intronic	14	54985916	CGRRF1	+
DEL	exonic	14	94980860	ADSSL1, AHNAK2, AK7, AKT1, AMN, ANKRD9, APOPT1, ASPG, ATG2B, BAG5, BCL11B, BDKRB1, BDKRB2, BEGAIN, BRF1, BTBD6, C14orf129, C14orf132, C14orf177, C14orf180, C14orf2, C14orf49, C14orf79, C14orf80, CCDC85C, CCNK, CDC42BPB, CDCA4, CINP, CKB, CLMN, CRIP1, CRIP2, CYP46A1, DEGS2, DICER1, DIO3, DLK1, DYNC1H1, EIF5, EML1, EVL, EXOC3L4, GLRX5, GPR132, GSC, HHIPL1, HSP90AA1, INF2, JAG2, KIAA0284, KIF26A, KLC1, MARK3, MOK, MTA1, NUDT14, PACS2, PAPOLA, PLD4, PPP1R13B, PPP2R5C, RCOR1, RD3L, RTL1, SERPINA3, SERPINA4, SERPINA5, SETD3, SIVA1, SLC25A29, SLC25A47, TCL1A, TCL1B, TDRD9, TECPR2, TEX22, TMEM121, TMEM179, TNFAIP2, TRAF3, TRMT61A, VRK1, WARS, WDR20, WDR25, XRCC3, YY1, ZBTB42, ZFYVE21, ZNF839	+		14	106501796		+
ITX	intergenic	14	40048709	FBXO33, NONE	-	exonic	14	37736275	MIPOL1	+
ITX	intronic	14	75181030	FCF1	-	intronic	14	30217685	PRKD1	+
ITX	intergenic	14	96080377	GLRX5, TCL6	-	intergenic	14	97774402	VRK1, LOC100129345	+
ITX	intergenic	14	44036682	LRFN5, FSCB	-	intronic	14	33550297	NPAS3	+
CTX	intronic	14	103961410	MARK3	+	intergenic	8	29261298	DUSP4, LINC00589	+
CTX	intergenic	14	101626204	MEG9, DIO3OS	-	intergenic	8	29259972	DUSP4, LINC00589	+
ITX	intronic	14	33669555	NPAS3	+	intergenic	14	96102422	GLRX5, TCL6	-
ITX	intronic	14	30303422	PRKD1	+	intergenic	14	76823138	RNU6-6, ESRRB	-
INS	intergenic	14	36699950	PTCSC3, MBIP	+	intronic	14	31813507	HEATR5A	+
INS	intronic	14	94980671	SERPINA12	+	intronic	14	64178259	SGPP1	+
INS	intergenic	14	64284711	SGPP1, SYNE2	+	intergenic	14	25646595	STXBP6, NOVA1	+
ITX	intergenic	14	62413909	SNAPC1, SYT16	-	intronic	14	61928728	PRKCH	+
ITX	intergenic	14	97705108	VRK1, LOC100129345	+	intergenic	14	97800472	VRK1, LOC100129345	-
DEL	intronic	15	49330104	SECISBP2L	+		15	49331173		+
CTX	intronic	15	45352742	SORD	-	intronic	6	62717796	KHDRBS2	+
ITX	intronic	17	80908606	B3GNTL1	-	intronic	17	80889762	TBCD	+
ITX	exonic	17	76157299	C17orf99	+	exonic	17	76183055	TK1	-

SV	Breakpoint A					Breakpoint B				
	Type	Chromosome	Position	Genes involved	Orientation	Type	Chromosome	Position	Genes involved	Orientation
DEL	exonic	17	76972849	C1QTNF1, CANT1, ENGASE, LGALS3BP, RBFOX3	+		17	77187934		+
ITX	intronic	17	76494447	DNAH17	+	intronic	17	76494976	DNAH17	-
ITX	intergenic	17	223006	RPH3AL, C17orf97	+	UTR3	17	416857	VPS53	-
ITX	intergenic	18	4978337	DLGAP1, C18orf42	-	intergenic	18	4951936	DLGAP1, C18orf42	+
ITX	intergenic	21	10461255	TEKT4P2, TPTE	+	intergenic	21	10462442	TEKT4P2, TPTE	-
DEL	exonic	22	43871864	ADM2, ALG12, ARHGAP8, ATXN10, BRD1, C22orf26, C22orf40, CELSR1, CERK, CRELD2, EFCAB6, FAM116B, FAM118A, FAM19A5, FBLN1, GRAMD4, GTSE1, HDAC10, IL17REL, KIAA0930, KIAA1644, LDOC1L, MAPK11, MAPK12, MLC1, MOV10L1, MPPED1, NUP50, PANX2, PARVB, PARVG, PHF21B, PIM3, PKDREJ, PLXNB2, PNPLA3, PNPLA5, PPARA, PPP6R2, PRR5, PRR5-ARHGAP8, RIBC2, SAMM50, SBF1, SELO, SMC1B, SULT4A1, TBC1D22A, TRABD, TRMU, TTC38, TUBGCP6, UPK3A, WNT7B, ZBED4	+		22	50921137		+
ITX	intronic	22	49008403	FAM19A5	-	intronic	22	43863917	MPPED1	+
CTX	UTR3	22	26847961	HPS4	+	intergenic	16	86598294	FLJ30679, FOXC2	+
DEL	intronic	22	31625522	LIMK2	+		22	31631326		+
INS	intronic	22	31656585	LIMK2	+	intronic	22	31587084	RNF185	+
ITX	intronic	22	50517359	MLC1	-	intronic	22	50183297	BRD1	+
INS	intergenic	22	33646135	SYN3, LARGE	+	intergenic	22	33619659	SYN3, LARGE	+
CTX	intergenic	X	146714168	MIR514A3, FMR1-AS1	+	intergenic	5	172827692	STC2, LOC285593	-

Table S4: List of structural variants (SVs) found in a metastatic MPTT.
Location of SVs are based on GRCh37/hg19. All somatic SVs (found only in tumor tissue) are shown, including: inter-chromosomal translocations (CTX), deletions (DEL), insertions (INS), and intra-chromosomal translocations (ITX).

Primer	Primer Sequence 5' → 3'	Amplicon size (bp)
ALPK1_F	GTTTTCCCAGTCACGACAGATCTTTCAAGCATTTCTGCAT	396
ALPK1_R	CAGGAAACAGCTATGACACAGGGACAACACACAAGGAT	
SH3RF1_F	GTTTTCCCAGTCACGACGGGATGCACACAACTATGAACA	399
SH3RF1_R	CAGGAAACAGCTATGACGCTCTGCAGATCTTTTGAGCTA	
PIAS2_F	GTTTTCCCAGTCACGACGACTGAGAATGCCCAGTGGT	357
PIAS2_R	CAGGAAACAGCTATGACCCTTTACCCGAAGCTTTCAA	
KLHDC5_F	GTTTTCCCAGTCACGACAGCAGCAGATGGTGTCTGTG	381
KLHDC5_R	CAGGAAACAGCTATGACTTCAATTCAGTCATGTTTCTGCTT	
BAP1_F	GTTTTCCCAGTCACGACGGAAGACGAGCCCAGAGG	386
BAP1_R	CAGGAAACAGCTATGACGTCCGGCAGGGAGAAAAG	
CENPL_F	GTTTTCCCAGTCACGACGCCAATGAGGAATATAGGACTTG	356
CENPL_R	CAGGAAACAGCTATGACGGGACACAAAAAGCATACCTG	
TTN_F	GTTTTCCCAGTCACGACTGTCCTGAGAGTGAGAGCAGTC	387
TTN_R	CAGGAAACAGCTATGACCACGGACACTCTTGGGAACT	
MORF4L2_F	GTTTTCCCAGTCACGACAACCATGGCTTGTTGAGGAC	353
MORF4L2_R	CAGGAAACAGCTATGACTTTCTCATCAAGGGGCGTAT	
MCF2_F	GTTTTCCCAGTCACGACCCTCACACGTATACATTTATGCAA	449
MCF2_R	CAGGAAACAGCTATGACCCCGCCAAAACATGACTATT	
DDI1_F	GTTTTCCCAGTCACGACACGGTCCCTACCTACCAAGTC	400
DDI1_R	CAGGAAACAGCTATGACGTCTACACGAGGCTGGTTCG	
KIFC1_F	GTTTTCCCAGTCACGACTCACGCAGCCACAGTGTATT	396
KIFC1_R	CAGGAAACAGCTATGACAGCACTACCACCCAGAGAGTTC	
LGALS3BP_F	GTTTTCCCAGTCACGACTGGAATATCCTCCGTGGTTC	384
LGALS3BP_R	CAGGAAACAGCTATGACCCTTTGTGTGCTTCATGTGC	
RFX4_F	GTTTTCCCAGTCACGACTGGAATTTCTTTCCCCTCAA	384
RFX4_R	CAGGAAACAGCTATGACATAAAGTCTTCTGGGGGATGG	

Table S5: Dideoxy sequencing primers.
Sequences of primers used for PCR and subsequent targeted dideoxy sequencing of mutational calls. M13 tags are highlighted in red for each primer.

Normal hair sheath	cSCC	MPTT
AGRN	AJUBA	ADAD2
ALAD	ATP1A1	ALPK1
ANGPTL2	BBS9	ANKRD20A1
ANGPTL7	CACNA1C	ANKRD36B
ANKH	CASP8	ASPM
CABLES1	CDKN2A	BAP1
CD34	CLCN3	C2ORF42
CD200	COBLL1	CENPL
COL17A1	CRY1	CLCN4
CRIM1	DCLK1	CST7
CXCL14	DCLRE1A	DCAF12L1
DAPK2	EIF2D	DDI1
DKK3	FAT1	EXT2
FGF18	FBXO21	FTH1
FOXC1	FLNB	GLRA1
FZD1	GLIS3	IRS2
GPC4	GRHL2	KIFC1
GLI2	HERC6	KLHL42
IL11RA	HRAS	LAT
IRX5	KMT2C	LGALS3BP
KCNK2	KMT2D	LUZP1
KRT5	LCLAT1	MAP1LC3A
LRG5	MAP3K9	MCF2
LHX2	MAPK1IP1L	MINOS-NBL1
LTBP2	NOTCH1	MORF4L2
MOXD1	NOTCH2	NBEAL2
NFATC1	OPN3	NBPF11
NFIB	PARD3	NDUFAF7
PLA2G4A	PEG10	NLRP12
PTPRK	PTEN	OPN1LW
RUNX1	RASA1	OR2M4
SARDH	RBM46	PARP4
SDC1	SEC31A	PDE1A
SEMA3E	SF3B1	PIAS2
SLC1A4	SNX25	PIK3CA
SOX9	TMEM51	POTEM
SSFA2	TP53	RFX4
TBX1	TRAPPC9	RLN2
TGFB2	VPS41	SH3RF1
TGFBI	WHSC1	TMEM232
TNC	ZNF644	TMPRSS11A
VDR		TTN
WIF1		USP17L20

Table S6: List of normal outer root sheath genes and those mutated in cutaneous SCC and MPTT. Lists of genes normally found to be active in the outer root sheath/bulb of hair or mutated in cutaneous SCC (cSCC) and MPTT.
